# Identifying unilateral aldosterone-producing adenomas using published algorithms and imaging: a systematic review and meta-analysis

**DOI:** 10.1530/EC-25-0339

**Published:** 2025-11-04

**Authors:** Elisabeth Ng, Stella May Gwini, Winston Zheng, Peter J Fuller, Jun Yang

**Affiliations:** ^1^ Centre for Endocrinology & Reproductive Health, Hudson Institute of Medical Research, Clayton, Australia; ^2^ Department of Endocrinology, Monash Health, Clayton, Australia; ^3^ Department of Molecular and Translational Science, Monash University, Clayton, Australia; ^4^ School of Public Health and Preventive Medicine, Monash University, Melbourne, Victoria, Australia; ^5^ Department of Medicine, Monash University, Clayton, Australia

**Keywords:** primary aldosteronism, subtyping, unilateral, adrenal vein sampling, systematic review, meta-analysis

## Abstract

**Objective:**

Primary aldosteronism (PA) caused by a unilateral aldosterone-producing adrenal adenoma is potentially curable by surgery. Radiologically visible adrenal adenomas are not necessarily functional, so adrenal vein sampling (AVS) is recommended to lateralise aldosterone excess. However, AVS is technically challenging and limited in availability. We sought to evaluate the diagnostic accuracy of published algorithms for lateralisation without AVS and identify the top-performing algorithms with the highest specificity.

**Design:**

Systematic review and meta-analysis.

**Methods:**

Algorithms to predict unilateral PA and enable lateralisation without AVS, using AVS with or without surgical outcomes as the comparator, were systematically evaluated. Meta-analysis and meta-regression were conducted to obtain and compare aggregated estimates, respectively.

**Results:**

There were 43 studies evaluating 30 unique algorithms grouped into four categories: i) those combining biochemical, radiological and demographic characteristics; ii) algorithms involving confirmatory testing or adrenocorticotropic hormone stimulation; iii) anatomical imaging; and iv) functional imaging. The meta-analysis demonstrated the highest specificity (95%) in the first two categories. The algorithm with the highest specificity (98%) for unilateral PA consisted of an adrenal nodule on CT, plasma aldosterone concentration >20.0 ng/dL (554 pmol/L), hypokalaemia (≤3.5 mmol/L) and renin concentration ≤5 mIU/L. Anatomical imaging for subtyping had poor specificity (69%), while functional imaging had higher specificity (86%), noting variation in functional imaging modalities and criteria for lateralisation.

**Conclusions:**

Algorithms may be used to identify unilateral PA without invasive testing in a modest proportion of patients. Validation in different populations is required to enable the widespread use of these tools.

**Significance statement:**

PA is gaining recognition as an important cause of hypertension and cardiovascular disease. The bottleneck created by AVS to subtype PA leads to delays in diagnosis and treatment. Selecting patients likely to have a unilateral aldosterone-producing adrenal adenoma, who can bypass AVS, will facilitate prompt surgical treatment and optimize the use of healthcare resources. Incorporating algorithms into the diagnostic evaluation of PA could streamline patient care and expedite appropriate treatments that will minimise the sequelae of aldosterone excess.

## Introduction

Primary aldosteronism (PA), the most common endocrine cause of hypertension, is under-recognised despite being treatable or potentially curable (1). The diagnostic process can be lengthy and complex due to the need for a screening blood test, confirmatory testing, and subtyping to determine the laterality of aldosterone excess (2). Determining if PA is caused by one adrenal gland or both is important as unilateral disease can be cured by adrenalectomy, while bilateral disease requires long-term treatment with mineralocorticoid receptor antagonists.

Adrenal vein sampling (AVS) is currently the gold standard method of subtyping PA. This involves cannulation of both adrenal veins to measure aldosterone and cortisol concentrations. AVS is resource-intensive, invasive, and limited in availability (3). Adrenal anatomical imaging that demonstrates a unilateral adenoma in the context of confirmed PA is not adequately specific to directly proceed to adrenalectomy due to the possibility of a non-functioning adrenal incidentaloma (4, 5). However, the presence of an adenoma together with other clinical or biochemical features may indicate a unilateral aldosterone-producing adenoma (APA). Multiple algorithms have been described to predict unilateral PA for the purpose of bypassing AVS and proceeding directly to surgery. An ideal algorithm needs to be highly specific to avoid false positive findings that could lead to inappropriate surgery. The aim of this systematic review and meta-analysis is to identify the top-performing algorithms which can identify unilateral PA with high specificity, to facilitate prompt referral for surgery without necessarily awaiting AVS.

## Methods

### Search strategy and selection criteria

**Figure 1:**
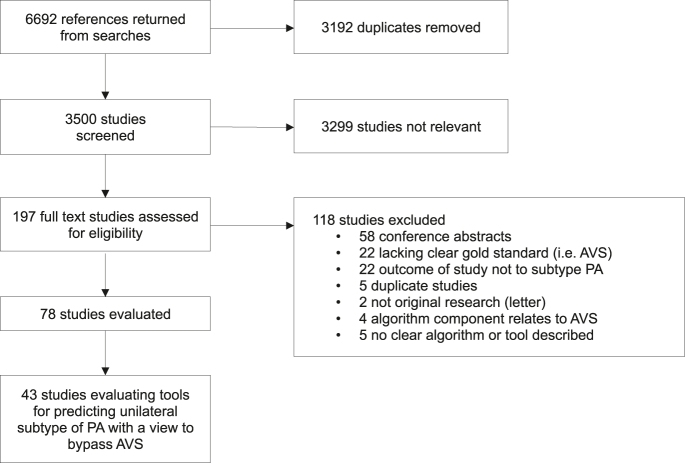
Study selection – preferred reporting items for systematic reviews and meta analyses (PRISMA) flow chart.

This systematic review and meta-analysis was performed and reported in accordance with the Preferred Reporting Items for Systematic Reviews and Meta-Analysis guidelines (6). PROSPERO registration was completed before project commencement (CRD42021277841). Medline and EMBASE were searched, from inception to 2 April 2024. The terms ‘hyperaldosteronism’ or ‘PA’ or ‘Conn syndrome’ with similar words were combined with keywords for the outcome, including ‘unilateral’, ‘bilateral’, ‘lateralisation’, ‘localisation’ and ‘subtype’. This was limited to papers published in English. Search terms can be found in Supplementary Table S1 (see the section on Supplementary materials given at the end of the article). Original studies were included if they evaluated any algorithm for subtyping PA, making comparison to a reference standard of AVS with or without surgical specimen histopathology. Studies were excluded if the tool or algorithm involved parameters from AVS, if aspects of the tool were unclear, or if the study design was not intended to predict subtype. This systematic review includes all studies evaluating an algorithm for determining a high likelihood of unilateral PA, where the intention was stated to be to bypass AVS (Fig. 1). All algorithms were required to include imaging to identify the side (right or left) that would be targeted for surgery. A separate systematic review describes the tools predicting unilateral PA which were intended to inform selection for AVS (7). Two independent investigators screened all studies meeting inclusion criteria and reviewed full text articles. Discrepancies at both the abstract and full text screening stage were resolved by a third independent investigator. To ensure consistency and accuracy in data extraction, both primary investigators jointly reviewed ten studies to establish a standardised approach for data interpretation and extraction. Full text data extraction was performed for all studies by one investigator, with a second investigator independently reviewing 20% of the studies. No discrepancies requiring third reviewer intervention arose at this stage, as there was no conflicting interpretation of data.

### Data analysis

The data extracted included study type, authors, country, participant clinical and demographic data, pathology results (aldosterone, renin, aldosterone-to-renin ratio (ARR), potassium, creatinine, and estimated glomerular filtration rate (eGFR)), confirmatory testing, CT imaging, AVS and histopathology, details of the algorithm or tool used, and figures used to calculate sensitivity, specificity, positive predictive value (PPV) and negative predictive value (NPV), where available and author conclusions. In the reporting of plasma aldosterone concentration (PAC) and ARR, conversion of PAC from ng/dL to pmol/L utilised a conversion factor of 27.74, and conversion from plasma renin activity as ng/mL/h to direct renin concentration (DRC) as mU/L utilised a factor of 8.2 (2).

Algorithms from the systematic review were included in a meta-analysis if they had sufficient data, and the diagnosis of unilateral PA when validated by AVS utilised a strict criterion of a lateralisation index (LI) >4. As many studies reported multiple algorithms, the one included in the meta-analysis was the one favoured by the authors as having the best performance (highest diagnostic accuracy). Algorithms that were not specifically favoured by authors were included if they were externally validated by an independent study. For single-centre studies with both development and validation cohorts, only the validation cohort data were used for analysis. A bivariate random effects meta-analysis model was employed. Sub-group meta-analyses were conducted when an algorithm was tested in at least three cohorts. Meta-regression was also conducted to examine whether there were any algorithm or study characteristics that were associated with the predictive accuracy of algorithms. The meta-analyses and meta-regression were conducted using the command metadta (8) from Stata Statistical Package version 17 (StataCorp. 2021. Stata Statistical Software: Release 17. USA: StataCorp LLC). A *P*-value <0.05 was considered statistically significant.

The Quality Assessment of Diagnostic Accuracy Studies-2 tool was used to assess the risk of bias (9). The proposed algorithm or tool was considered the index test and the gold standard test was considered the reference standard. Where data collected during the work-up of PA was used in the index test, an appropriate interval between the index test and reference standard (AVS) was assumed. Risk of bias and applicability concerns were stated to be unclear where relevant information was not provided. High risk of bias and applicability concerns were documented for studies where the AVS LI for determining unilateral PA was <4.

## Results

There were 43 studies included in this review (Fig. 1), evaluating 30 unique algorithms or tools intended to indicate a high likelihood of unilateral PA. Of these, 20 were tested in multiple cohorts and 13 had variations in criteria, e.g. different cut-offs using the same scoring system or different methods of analysis using the same form of imaging. The 30 unique tools were derived from 134 tools in total (Supplementary Table S3), differing by either cohort (training and validation), algorithm parameters or variation in scores applied to the same algorithm. There were 49 unique patient cohorts from the 43 studies.

### Study characteristics

The 43 studies were published between 1985 and 2024 (two in 2024, three in 2023, seven in 2022, four in 2021, and six in 2020). Most were single-centre studies (34/43) from referral centres, the majority in Europe and China. Screening for PA was most commonly performed by ARR and confirmed by saline suppression testing (SST). The study cohorts are described in Supplementary Table S2.

Examination of the 30 algorithms or tools indicated they could be grouped into four categories based on the parameters used to define the algorithms, i.e. tools combining biochemical, radiological and demographic characteristics (without confirmatory testing), those involving confirmatory testing and ACTH stimulation testing, anatomical imaging alone and functional imaging.

### Algorithms combining biochemical, radiological and demographic characteristics (without confirmatory testing)

There were 17 studies reporting on 15 unique algorithms tested in 20 unique cohorts in this category. A specificity of 100% was reported for multiple tools (12 tools from ten studies tested in 11 cohorts) but most were not externally validated after evaluation in small studies. Six had 100% specificity in more than one cohort (Table 1); these included a combination of hypokalaemia, eGFR, unilateral adenoma on CT, urinary aldosterone, age, PAC and DRC (10, 11, 12, 13, 14). A specificity above 90% was reported by eight studies for seven unique algorithms (Table 1) (10, 11, 13, 14, 15, 16, 17).

**Table 1:** Algorithms with >90% specificity reported in more than one cohort.

Algorithm components	Author (year) and cohort	Criteria used to identify unilateral PA	Number with PA based on AVS (unilateral/bilateral)	Sensitivity	Specificity	PPV	NPV
**Algorithms combining biochemical, radiological and demographic characteristics**							
Küpers *et al*. (11) 1) Typical adrenal nodule and normal contralateral gland on CT imaging (3 points) 2) Serum potassium <3.5 mEq/L (2 points) 3) eGFR (mL/min/1.73 sqm) (MDRD) < 80 (0 points); 80–99 (1 point), ≥100 (2 points)	Küpers (2012)	Score ≥5	49/38	0.531	**1.000**	1.000	0.617
	Song (2022) validation cohort	Score 5 using unilateral nodule ≥8 mm and eGFR ≥100	84/117	0	**1.000**	0	0.582
	Song (2022) validation cohort	Score ≥5 using unilateral nodule ≥8 mm, serum potassium <3.5 mmol/L, eGFR ≥100	84/117	0	**1.000**	0	0.582
	Venos (2014)	Score ≥6	64/46	0.391	0.957	0.926	0.530
	Song (2022) development cohort	Score ≥5 using unilateral nodule ≥8 mm, serum potassium <3.5 mmol/L, eGFR ≥100	268/88	0.269	0.932	0.923	0.295
	Song (2022) validation cohort	Score ≥5 using unilateral nodule ≥8 mm and serum potassium <3.5 mmol/L	84/117	0.262	0.983	0.917	0.650
Young *et al.* (57) 1) Age ≤40 years 2) Unilateral adenoma on CT ≥10 mm	Küpers (2012)	Both criteria met	49/38	0.184	**1.000**	1.000	0.487
	Kolosova (2022) development cohort	Both criteria met	96/54	0.125	**1.000**	1.000	0.391
	Rossi (2022)	Both criteria met	128/100	0.727	0.950	0.949	0.731
	Song (2022) development cohort	Both criteria met	268/88	0.209	0.943	0.918	0.281
	Song (2022) validation cohort	Both criteria met	84/117	0.060	0.966	0.556	0.591
Endocrine Society guidelines (Funder *et al.* (2)) 1) Spontaneous hypokalaemia (<3.5 mmol/L) 2) PAC >30 ng/dL (831 pmol/L) 3) Unilateral nodule >10 mm and normal-appearing contralateral adrenal 4) Age <35 years	Sam (2022)	All criteria met	152/119	0.026	**1.000**	1.000	0.446
	Song (2022) development cohort	All criteria met	268/88	0.071	**1.000**	1.000	0.261
	Song (2022) validation cohort	All criteria met	84/117	0.012	**1.000**	1.000	0.585
Modified Küpers’ score by Zhang *et al.* (14) 1) Hypokalaemia <3.5 mmol/L (2 points) 2) Urinary aldosterone level (μg/day) – <13 (0 points); 13–19 (1 point); 19–23 (2 points); >23 (3 points) 3) Typical Conn’s adenoma on CT (3 points)	Zhang (2017)	Age <40 years AND score ≥6	64/84	0.235	**1.000**	1.000	0.500
	Song (2022) development cohort	Age <40 years AND score ≥6	268/88	0.049	**1.000**	1.000	0.257
Song *et al.* (13) 1) Serum potassium ≤3.5 mmol/L 2) PAC ≥20 ng/dL (554 pmol/L) 3) Plasma renin concentration ≤5 mIU/mL 4) Unilateral adrenal nodule ≥10 mm on CT	Song (2022) development cohort	All criteria met	268/88	0.522	**1.000**	1.000	0.407
	Song (2022) validation cohort	All criteria met	84/117	0.155	**1.000**	1.000	0.622
Umakoshi *et al.* (45) 1) PAC >15.9 ng/dL (440 pmol/L) 2) Serum potassium <3.5 mmol/L 3) Unilateral nodule >10 mm 4) Age <35 years	Song (2022) validation cohort	All criteria met	84/117	0.012	**1.000**	1.000	0.585
	Kolosova (2022) development cohort	All criteria met	96/54	0.042	**1.000**	1.000	0.370
	Umakoshi (2018)	All criteria met	256/102	0.105	0.971	0.900	0.302
	Song (2022) development cohort	All criteria met	268/88	0.119	0.977	0.941	0.267
Rossi *et al.* (16) 1) Hypokalaemia with serum potassium <3.6 mEq/L 2) Unilateral adrenal nodule on CT 3) Age ≤45 years	Rossi (2022)	All criteria met	128/100	0.531	**1.000**	1.000	0.625
	Kolosova (2022) development cohort	All criteria met	96/54	0.135	0.981	0.929	0.390
	Song (2022) development cohort	All criteria met	268/88	0.340	0.932	0.938	0.317
	Song (2022) validation cohort	All criteria met	84/117	0.048	0.991	0.800	0.592
Lee *et al.* (15) 1) Serum potassium <3.5 mmol/L 2) PAC >30 ng/dL (831 pmol/L) 3) Unilateral lesion on CT >7 mm	Lee (2021)	All criteria met	309/157	0.405	0.911	0.899	0.437
	Song (2022) development cohort	All criteria met	268/88	0.489	0.955	0.970	0.380
	Song (2022) validation cohort	All criteria met	84/117	0.190	**1.000**	1.000	0.632
	Kolosova (2022) development cohort	All criteria met	96/54	0.375	0.926	0.900	0.455
Zhang *et al.* (14) 1) Hypokalaemia <3.5 mmol/L (2 points) 2) Urinary aldosterone concentration (μg/day) – <13 (0 points); 13–19 (1 point); 19–23 (2 points); >23 (3 points) 3) Typical Conn’s adenoma on CT (3 points)	Zhang (2017)	Age <40 years AND score ≥5	64/84	0.453	0.905	0.784	0.685
	Song (2022) development cohort	Age <40 years AND score ≥5	268/88	0.351	0.977	0.979	0.331
**Algorithms involving confirmatory testing alone or combined with biochemical, radiological and demographic characteristics**							
Holaj *et al.* (21) Adrenal nodule = 2 points PAC after saline suppression test ≥165 ng/L (457 pmol/L) = 1 point	Holaj (2022) development cohort	Score of 3 points	96/54	0.479	**1.000**	1.000	0.519
	Kolosova (2022) validation cohort	Score of 3 points	94/44	0.362	**1.000**	1.000	0.423
SPACE score by Burrello *et al.* (18) 1) PAC at screening: >25 ng/dL (693 pmol/L) (0.5 points) 2) Lowest potassium (mEq/L): <3.4 (5 points), 3.4–3.9 (1.5 points) 3) PAC post-confirmatory test (ng/dL): 15.1–19.9 (1 point), ≥20 (554 pmol/L) (2 points) 4) Nodule at CT scanning (4 points) 5) Largest nodule at CT scanning (diameter, mm) 11–30 (1 point) >30 (2 points) 6) Adrenal CT scan: bilaterally abnormal (4.5 points), unilateral abnormality (6.5 points)	Burrello (2020) training cohort	SPACE score >16	93/57	0.473	0.983	0.978	0.533
	Burrello (2020) validation cohort	SPACE score >16	40/25	0.375	0.920	0.882	0.479
	Kocjan (2022)		59/85	0.424	0.918	0.781	0.697
Random forest (RF) model by Burrello *et al.* (18) Six selected variables used: 1) Unilateral nodule present or not (8 mm) 2) Lowest potassium ≤ or >3.9 mmol/L 3) PAC post-confirmatory test ≤ or >8.9 ng/dL (247 pmol/L) 4) Nodule diameter ≤ or >14 mm 5) PAC at screening ≤ or >30.3 ng/dL (839 pmol/L)	Song (2022) development cohort	Diagnostic classification tree shown in Burrello *et al.* (18)	268/88	0.459	0.989	0.992	0.375
	Song (2022) validation cohort	Diagnostic classification tree shown in Burrello *et al.* (18)	84/117	0.095	0.992	0.889	0.604
	Kolosova (2022) development cohort	Diagnostic classification tree shown in Burrello *et al.* (18)	96/54	0.396	1.000	1.000	0.482
**Anatomical imaging**							
MRI	Ladurner (2017)	Adrenal adenoma seen on MRI	81/6	0.704	**1.000**	1.000	0.200
	Sohaib (2000)	Adrenal adenoma seen on MRI	10/10	0.460	**1.000**	1.000	0.703
**Functional imaging**							
^ 11^C-metomidate PET/CT	Burton (2012)	LI >1.25 and tumour SUVmax >17	25/10	0.640	**1.000**	1.000	0.591
	Wu (2023)	LI >1.25 to achieve PASO complete or partial biochemical success	74/42	0.743	0.952	0.965	0.678

Bold indicates 100% specificity. Abbreviations: CT, computed tomography; eGFR, estimated glomerular filtration rate; MRI, magnetic resonance imaging; PAC, plasma aldosterone concentration; PASO, primary aldosteronism surgical outcome; LI, lateralisation index.

### Algorithms involving confirmatory testing or ACTH stimulation testing

Eight studies reported on eight unique algorithms tested in 13 unique cohorts in this category (10, 18, 19, 20, 21, 22, 23). Confirmatory tests used were SST (three studies (10, 21, 22)), captopril challenge test (CCT) or SST (three studies (13, 18, 19)), frusemide upright test or CCT (one study (23)) and oral salt loading (one study (20)). A specificity over 90% was reported for seven tools in eight studies, three of which demonstrated high specificity in multiple cohorts. The algorithm by Holaj *et al.* involving adrenal imaging and PAC post-SST ≥16.5 ng/L (457 pmol/L) was the only one of four algorithms with 100% specificity that underwent external validation; specificity was consistent at 100% but sensitivity was lower at 36% (compared to 48% in the development cohort) (10, 21). The two algorithms that demonstrated high specificity (90–99%) in multiple cohorts were both by Burrello *et al.* (SPACE score and random forest (RF) model) (18). The SPACE score comprised PAC, lowest potassium, PAC post-confirmatory test (SST or CCT) and CT findings (nodule presence and size). This had a 71–92% specificity and 38–88% sensitivity across three cohorts (from Italy, Germany and Slovenia) (18, 22). The RF model used to develop the SPACE score (18) had 99% specificity in cohorts from China and Australia, applying the combination of a unilateral nodule ≥8 mm, hypokalaemia <3.9 mmol/L, PAC post-CCT or SST >8.9 ng/dL (247 pmol/L) and screening PAC >30.3 ng/dL (841 pmol/L) (13).

One algorithm involving ACTH stimulation (PAC >77.90 ng/dL (2,161 pmol/L) 2 h post-ACTH) reported a high specificity but has not been externally validated (Supplementary Table S3) (24).

### Anatomical imaging alone

Adrenal CT or MRI to subtype PA was studied in 14 studies (14 unique cohorts). CT alone for subtyping had a specificity of 22–93% and sensitivity of 35–100% across ten cohorts (Supplementary Table S3) (15, 25, 26, 27, 28, 29, 30, 31, 32). A specificity of 93% was reported in one study that strictly looked for a definite nodule ≥10 mm with unequivocally normal contralateral gland morphology (27). All other studies reported a specificity <78% for the detection of unilateral PA. Li *et al.* and Zhang *et al.* evaluated the left to right adrenal volume ratio (L/Rv) as a tool to indicate a higher likelihood of unilateral PA, with Zhang *et al.* additionally using the difference between the left and right adrenal volume (L/Rv) (33, 34). Optimal cut-offs in their cohorts had specificities of 100% and 96–97%, respectively, however, neither tool has been externally validated (33, 34). Two studies evaluating the accuracy of MRI reported 100% specificity but sample sizes were small (26, 35).

### Functional imaging

Functional imaging using ^11^C-metomidate PET/CT, ^68^Ga-Pentixafor PET/CT or ^131^I-iodocholesterol (NP59) imaging were evaluated in ten studies (ten cohorts) (31, 36, 37, 38, 39, 40, 41, 42, 43, 44). Various cut-offs were used in studies of NP-59 and ^68^Ga-Pentixafor PET/CT, while for ^11^C-metomidate PET/CT, a LI >1.25 was evaluated in multiple studies. All studies evaluated patients with PA and a visible unilateral nodule.

NP-59 imaging was assessed in three small studies, all of which were at high risk of bias (Supplementary Fig. S1B) (31, 38, 41). A specificity of 100% was reported for a LI >2.55 (maximum count on the high value side divided by the low value side) (41), and in another study, a normal-to-adenoma ratio of ≤0.5 3–4 days after NP59 (38).

^68^Ga-Pentixafor PET/CT has also been reported to achieve 100% specificity using various PET imaging parameters in three different studies. Hu *et al.* found 100% specificity using a LI >1.65 at 10 min (sensitivity 77%) or LI >3.15 at 40 min (sensitivity 44%) in 100 patients with PA (39). Yin *et al.* reported 100% specificity for a LI >1.39 (sensitivity 90%) or lesion maximum standardized uptake value (SUVmax) >5.71 (sensitivity 79%). Ding *et al.* reported 100% specificity in 29 patients when applying a lesion-to-liver ratio ≥2.36 (100% sensitivity) or SUVmax ≥11.18 (88% sensitivity), while a LI ≥2.12 had a specificity of 93% and a sensitivity of 100% (37).

^11^C-Metomidate PET/CT was tested in four studies; a specificity of 100% was observed in a study of 35 participants based on an LI >1.25 (SUVmax in adrenal tumour divided by contralateral adrenal gland) and tumour SUVmax >17. Larger studies of ^11^C-metomidate in Singapore and the UK defining lateralisation as SUVmax ratio >1.25 had a specificity of 67–87% with a sensitivity of 74–82% (40, 43).

### Meta-analysis

The meta-analysis included 23 unique algorithms/tools from 26 studies. Algorithms achieving the highest specificity were those that combined biochemical, radiological and demographic characteristics, and those that involved confirmatory testing (Table 2). With regards to specificity, heterogeneity was high for all except the algorithm combining an adrenal nodule on CT with a PAC post-SST of ≥165 ng/L (457 pmol/L) (*I*^2^ = 0.1%) (21) and with age (<40 (10, 11, 13) or 46 years (16)) (*I*^2^ = 8.1%). Sensitivity was low to moderate in all categories and was the highest for functional imaging (72%), which also had the lowest heterogeneity for sensitivity (*I*^2^ = 23%) (Table 2). Specificity and sensitivity varied across tools (*P*-value <0.001 for both), with variation in spread shown in summary receiver operating characteristic plots (Supplementary Figs S1, S2, S3). The most specific algorithms were those by Song *et al.* combining a unilateral nodule on CT with hypokalaemia (≤3.5 mmol/L), elevated PAC (≥20 ng/dL (554 pmol/L)) and low DRC (≤5 mIU/L) and that by Umakoshi *et al.* combining hypokalaemia (<3.5 mmol/L), PAC >15.9 ng/dL (440 pmol/L), unilateral adenoma ≥10 mm on CT and age <35 years (13, 45). These two algorithms demonstrated the highest specificities (98% for Song *et al.* (13) and 97% for Umakoshi *et al.* (45)), with the algorithm by Song *et al.* showing superior sensitivity (33 vs 9%), making it potentially more useful in clinical practice.

**Table 2:** Random effects meta-analysis results for studies predicting unilateral PA, split according to algorithm components.

Algorithm category	Number of cohorts	Patients*	Sensitivity^†^		Specificity^†^	
			Estimate (95% CI)	*I* ^2^	Estimate (95% CI)	*I* ^2^
**Algorithms combining biochemical, radiological and demographic characteristics**	39	2,473	24% (17–33)	94.2%	95% (93–97)	62.5%
Algorithm by Song *et al.* (13), all of: 1) Serum potassium ≤3.5 mmol/L 2) Plasma aldosterone concentration (PAC) ≥20 ng/dL (554 pmol/L) 3) Plasma renin concentration ≤5 mIU/mL 4) Unilateral adrenal nodule ≥10 mm on CT	3	695	33% (18–53)	91.3%	98% (88–100)	55.9%
Algorithm by Umakoshi *et al.* (45), all of: 1) PAC >15.9 ng/dL (440 pmol/L) 2) Serum potassium <3.5 mmol/L 3) Unilateral nodule >10 mm on CT 4) Age <35 years	5	1,065	9% (5–17)	84.1%	97% (92–99)	60.0%
Both of (16) and (57): 1) Unilateral adenoma on CT ≥10 mm 2) Age ≤40 years or 45 years	4	794	15% (10–22)	57.3%	96% (93–98)	8.1%
Algorithm by Rossi *et al.* (16), all of: 1) Serum potassium <3.6 mEq/L 2) Unilateral adrenal nodule on CT 3) Age ≤45 years	3	707	15% (6–33)	89.1%	96% (91–99)	48.7%
Algorithm by Lee *et al.* (15), all of: 1) Hypokalaemia (serum potassium <3.5 mEq/L) 2) PAC >30 ng/dL (831 pmol/L) 3) Unilateral lesion on CT	4	1,173	36% (26–49)	87.0%	95% (88–98)	65.4%
Variations of prediction score by Küpers *et al.* (11): 1) CT imaging – typical adrenal nodule and normal contralateral gland (3 points) 2) Serum potassium <3.5 mEq/L (2 points) 3) eGFR (MDRD) mL/min/1.73 sqm – <80 (0 points); 80–99 (1 point), ≥100 (2 points)	11	1,305	34% (15–59)	96.0%	92% (82–96)	81.4%
**Algorithms involving confirmatory testing**	11	1,192	43% (31–55)	88.5%	94% (89–97)	57.5%
Excluding studies with baseline PAC as part of algorithm	8	1,034	42% (27–59)	93.3%	93% (86–97)	71.7%
SPACE model by Burrello *et al.* (18) – score >16	3	327	59% (30–83)	90.7%	85% (71–93)	67.5%
Random forest model by Burrello *et al.* (18)	5	707	33% (21–48)	91.3%	96% (91–98)	62.8%
**Anatomical imaging (CT or MRI)**	11	1,328	64% (52–75)	82.9%	69% (48–84)	73.5%
CT imaging	6	707	64% (50–76)	74.3%	51% (35–66)	47.2%
**Functional imaging**	5	412	72% (64–78)	22.7%	86% (63–96)	58.7%

Abbreviations: CI, confidence interval; CT, computed tomography; eGFR, estimated glomerular filtration rate; MRI, magnetic resonance imaging; PAC, plasma aldosterone concentration.

* Total number of patients with AVS results. In cases where multiple algorithms were tested on the same group of patients, the patients were only counted once.

^†^ Overall comparison across algorithms: *P*-value for sensitivity <0.001 and *P*-value for specificity <0.001.

Meta-regression was only conducted for the group of algorithms containing biochemical, radiological, and demographic characteristics, given the high number of studies in this subgroup and the variation in characteristics, which permitted evaluation of the effect of adding or removing certain algorithm characteristics. Table 3 shows that sensitivity of algorithms to select for unilateral PA was improved by inclusion of serum potassium without affecting specificity. Addition of eGFR significantly increased sensitivity, but at the expense of specificity. Examination of a Deek’s funnel plot for each group of algorithms did not indicate significant bias (Supplementary Fig. S4). In a sensitivity analysis, removing studies in the category combining biochemical, radiological, and demographic characteristics and the confirmatory testing category did not significantly alter specificity or sensitivity. For the anatomical imaging studies, overall specificity was sensitive to the removal of individual tools. Removing the tool by Zhang *et al.* (left-to-right adrenal volume ratio to predict lateralisation) (34) reduced the overall specificity of this category from 69 to 63%, and removing the data by Lau *et al.* using CT imaging to predict subtype (27) reduced specificity to 66%. Removing the studies by Lee *et al.* and Raman *et al.* improved overall specificity of this category to 72% (15, 29).

**Table 3:** Results from meta-regression of study and algorithm characteristics for algorithms combining biochemical, radiological and demographic characteristics.

Characteristic	Number of cohorts	Sensitivity			Specificity		
		Summary	Between group comparison		Summary	Between group comparison	
		Estimate (95%CI)	RR (95%CI)*	*P*-value	Estimate (95%CI)	RR (95%CI)*	*P*-value
**Algorithm specific**							
Inclusion of serum potassium							
No	8	9% (4–19)	Ref^†^		99% (96–100)	Ref^†^	
Yes	31	27% (17–38)	2.81 (1.45–5.44)	0.002	97% (94–98)	0.98 (0.96–1.00)	0.078
Inclusion of baseline aldosterone concentration							
No	23	25% (15–40)	Ref^†^		96% (92–98)	Ref^†^	
Yes	16	18% (9–33)	0.73 (0.32–1.65)	0.449	99% (96–99)	1.03 (0.99–1.06)	0.150
Inclusion of baseline eGFR							
No	27	17% (10–26)	Ref^†^		98% (97–99)	Ref^†^	
Yes	12	40% (25–58)	2.46 (1.52–3.97)	<0.001	91% (82–96)	0.93 (0.86–0.99)	0.029
**Study specific**							
Study country							
China	20	13% (8–22)	Ref^†^		99% (97–99)	Ref^†^	
Czech Republic	6	21% (8–44)	1.56 (0.56–4.35)	0.394	97% (90–99)	0.99 (0.95–1.02)	0.388
Other Europe	7	51% (26–76)	3.88 (1.82–8.27)	<0.001	89% (71–97)	0.91 (0.79–1.03)	0.138
Japan	3	31% (9–68)	2.36 (0.72–7.79)	0.158	94% (74–99)	0.95 (0.86–1.06)	0.362
Other	3	54% (20–85)	4.09 (1.68–9.97)	0.002	87% (54–97)	0.88 (0.70–1.11)	0.271

Abbreviations: CI, confidence interval; eGFR, estimated glomerular filtration rate; RR, relative ratio.

* Relative ratio compares the current level with the base level, which has RR = 1.00. The purpose is to statistically compare two sensitivity or specificity measures.

^†^ Ref: base level.

### Quality assessment

Most of the studies displayed moderate to high risk of bias, as assessed by the QUADAS-2 score (Supplementary Fig. S5). The highest concern of risk of bias and applicability related to the reference standard, in particular whether AVS interpretation correctly identified unilateral PA (Supplementary Fig. S5). With regards to the risk of bias with the index test (proposed algorithm), most algorithms were developed in cohorts with an AVS-defined subtype. The index tests did not involve AVS components, but their interpretation was informed by AVS results, meaning index test thresholds were not pre-specified. In this context, however, this lack of a pre-specified threshold is not considered a source of bias.

## Discussion

This systematic review evaluated published algorithms for identifying unilateral PA without AVS. A total of 43 studies were included, of which 26 were examined in the meta-analysis based on data availability and stringent lateralisation criteria. All algorithms required radiological evidence of an adrenal adenoma. The meta-analysis found that algorithms combining biochemical and radiological characteristics, with or without age, sex and confirmatory testing results, had the highest specificity for unilateral PA as confirmed by AVS, compared to other categories (Table 2). The algorithm with the highest specificity in the meta-analysis (98%) required the presence of a unilateral adrenal nodule >10 mm on CT imaging, PAC >20 ng/dL (554 pmol/L), serum potassium <3.5 mmol/L and DRC ≤5 mIU/L (13). However, its sensitivity varied between 16 and 52% in three cohorts from two studies, indicating the need for further validation before routine clinical use. Another algorithm, which incorporated PAC >15.9 ng/dL (440 pmol/L), serum potassium <3.5 mmol/L, a unilateral adrenal nodule >10 mm on CT and age <35 years, demonstrated a similarly high specificity (95%) but had lower sensitivity (9%) when tested in four cohorts (from three studies).

In selecting an algorithm to identify unilateral PA without AVS, priority is given to high specificity to ensure an appropriate referral for surgery. Whilst 100% specificity was reported by multiple algorithms or tools, most were small studies and/or had not been externally validated. Of these, the studies on NP-59 imaging and MRI imaging were at high risk of bias and tested in small cohorts, and are therefore not recommended for routine use to subtype PA. Most of the tools with high specificity that have been validated in multiple cohorts required a combination of biochemical, demographic and radiographic data, with the latter being CT imaging in all studies in this category. The combination of spontaneous hypokalaemia (<3.5 mmol/L), elevated PAC >30 ng/dL (830 pmol/L), CT-detected adenoma ≥10 mm and age <35 years is the recommended criteria for bypassing AVS in the Endocrine Society guidelines (2), and has mutual elements with the prediction score by Küpers *et al.* (11) The above combination with a PAC >30 ng/dL (830 pmol/L) had 100% specificity when evaluated in three cohorts from two studies but sensitivity was very low at 1–7%, making it less clinically useful (12, 13). Zhang *et al.* modified the prediction score by Küpers *et al.* to replace eGFR criteria with urinary aldosterone, and a score ≥6 had 100% specificity for unilateral PA in those <40 years in two cohorts but sensitivity was only 4–24%; lowering the cut-off to ≥5 lowered specificity to 91–98% but increased sensitivity to 35–45% (Table 1) (13, 14).

Younger age was a key factor in multiple algorithms for selecting patients with unilateral PA, with various age cut-offs applied, including 35 years, (11, 13, 33, 45), 40 years (11, 14, 46), 45 years (16) and 52 years (23) Heterogeneity in population characteristics, including age distribution and ethnicity, is likely to impact the prevalence of PA and of its subtypes. This is likely to affect the diagnostic accuracy of the algorithms in different populations. In most studies, cohorts had a median age ∼50 years, which may lead to uncertainty about the algorithms’ accuracy in older adults. Acknowledging variations in algorithm performance between different populations emphasises the importance of validation in diverse cohorts.

Nuclear imaging is an emerging non-invasive method of subtyping. Whilst it is not an algorithm per se, it is a tool that could provide an alternative to AVS and so has been included in this review. This review examined NP-59, ^68^Ga-PentixaFor PET/CT and ^11^C-metomidate PET/CT, with a focus on recent studies with reasonable sample sizes. A Spanish study of NP-59 imaging in 86 people with PA did not show high specificity, contrary to the smaller studies included in this review (47). ^11^C-metomidate PET/CT was recently shown to be non-inferior to AVS in 128 people with PA (43).^68^Ga-PentixaFor PET/CT has been reported to have high specificity using various PET-LI cut-offs in studies conducted in China, although the high prevalence of KCNJ5 mutations in the Chinese population should be considered (39, 44). These functional imaging methods show promise for identifying unilateral PA without AVS but require further validation in diverse cohorts and assessment of clinical feasibility.

The most typical presentation of a clear-cut unilateral adenoma causing PA (Conn’s syndrome) is more likely in people with the somatic KCNJ5 mutation. Carriers of this mutation are typically younger, female and East Asian, and they demonstrate higher PAC and hypokalaemia (48, 49). Efforts have been made to predict the KCNJ5 mutations using measurements of urinary hybrid steroids or plasma steroid profiling, as well as more comprehensive machine learning models (43, 50). In the future, these tools could be integrated with existing algorithms to enhance the identification of unilateral PA.

A limitation of this review and meta-analysis is the potential impact of lack of uniformity in the diagnostic steps to diagnose and subtype PA across studies, and variations in the assays used to measure PAC and renin (Supplementary Table S2). Strict criteria for the risk of bias assessment were used to highlight the studies of greatest concern regarding applicability, including where the AVS LI threshold <4 (Supplementary Fig. S5). Some of the algorithms were only applicable following a robust diagnosis of PA as the algorithm itself did not include markers of hyperaldosteronism (e.g. CT imaging and age without aldosterone or renin), while other algorithms could be applied following initial screening based on the patient’s aldosterone, renin, potassium and CT imaging. Most of the algorithms in the review were single centre studies, so we focused on algorithms which have been validated in at least one other cohort. The lack of perfect specificity and validation in multiple cohorts must be considered when applying these algorithms; the small chance of a false positive result leading to unnecessary surgery must be weighed against the benefits of bypassing AVS. Furthermore, these algorithms can achieve higher specificity than adrenal imaging alone and may therefore be highly valuable in low-resource settings, where decisions to operate are commonly based on adrenal CT (51).

Another limitation of our review is that the majority of studies relied on AVS for the subtyping outcome, as it was the standard of care at the time of publication. The criteria for confirming biochemical cure after surgery for unilateral PA were formally established by the Primary Aldosteronism Surgical Outcome (PASO) study, which was published subsequent to the majority of the studies evaluated here (52). Furthermore, most studies included in this review pre-date the availability of histopathological data incorporating CYP11B2 immunohistochemistry, which can provide additional information about the PA diagnosis and prognosis (53). Classical PA, defined by a solitary well-circumscribed CYP11B2-positive adenoma (53), is strongly associated with complete biochemical response and therefore reflects true unilateral disease. Conversely, non-classical PA (e.g. aldosterone-producing nodules or aldosterone-producing micronodules) is often associated with an absent biochemical response, suggesting underlying bilateral aldosterone excess despite apparent lateralisation on AVS (53). The lack of immunohistochemistry data and PASO outcomes to confirm unilateral PA makes it possible that some patients classified as unilateral PA in the reported studies may have had asymmetric bilateral PA (54). A further important limitation relates to the fact that people with lateralising disease without a discrete adrenal nodule may also be cured by adrenalectomy. Since these individuals would not be identified by the imaging-dependent algorithms examined in this review, these published algorithms may miss unilateral cases lacking a visible adenoma.

As most studies were retrospective or cross-sectional, a further limitation of this review is the lack of standardisation in how each study selected patients for PA screening, which likely reflected the local clinical practices. Region- and centre-specific differences in test thresholds and availability may have influenced the observed prevalence and characteristics of lateralised disease. Those with more severe disease may have been preferentially screened, leading to enrichment of lateralising forms of PA; a recent study showed higher rates of lateralisation in those with more severe PA (e.g. 73% in severe vs 15% in mild PA) (55). Awareness of PA has increased in recent years, but previous efforts to diagnose PA likely focused on those with more severe disease. The 2016 Endocrine Society guidelines on PA provided a limited set of criteria to prompt screening for PA (2), but recent guidelines are now recommending screening for PA in all hypertensive adults (56). As this broader screening approach is adopted, future cohorts will likely include a greater proportion of individuals with milder forms of PA. Consequently, the diagnostic performance of existing prediction algorithms, which were primarily developed and validated in patients with more pronounced disease, will need to be re-evaluated in this new context. Nonetheless, these tools remain relevant in current practice by helping to triage patients who could be considered for adrenalectomy without AVS, thereby reducing the burden on AVS services. For patients with milder or less distinct clinical presentations, where algorithms may not provide definitive guidance, AVS or alternative subtyping methods will still be indicated.

The utility of these algorithms for the individual patient will depend on the availability of components of the algorithm or tool used to indicate a higher likelihood of unilateral PA, and the risk–benefit discussion between the clinician and patient. Testing and validating existing algorithms would enable identification of the most appropriate centre-specific algorithm. In this study, the highest specificities were seen with the algorithm by Song *et al.* combining a unilateral nodule on CT with hypokalaemia (≤3.5 mmol/L), elevated PAC (≥20 ng/dL (554 pmol/L)) and low DRC (≤5 mIU/L) and that by Umakoshi *et al.* combining hypokalaemia (<3.5 mmol/L), PAC >15.9 ng/dL (440 pmol/L), unilateral adenoma ≥10 mm on CT and age <35 years (13, 45). For older patients, where the algorithms involving an age cut-off cannot be applied, the SPACE score >16 or RF model by Burrello *et al.* were highly specific, the latter involving a unilateral nodule on CT, serum potassium <3.9 mmol/L, PAC post-CCT or SIT >8.9 ng/dL (247 pmol/L) and PAC at screening >30.3 ng/dL (841 pmol/L) (13).

Identifying patients who can bypass AVS and proceed directly to adrenalectomy for a unilateral adrenal adenoma on imaging has the potential to simplify the diagnostic and treatment pathway for PA. This approach may increase clinician motivation to screen for the condition and, for patients with severe disease consistent with lateralising PA, reduce the burden of investigations and shorten the time to surgery. However, as awareness grows and more patients with milder disease are identified, these algorithms will need to be refined to improve sensitivity and ensure applicability across a wider clinical spectrum. Prospective validation in more diverse cohorts will be essential.

## Supplementary materials



## Declaration of interest

Jun Yang is a Senior Editor of *Endocrine Connections*. Jun Yang was not involved in the review or editorial process for this paper, on which she is listed as an author.

## Funding

JY is supported by an NHMRC Investigator Grant (APP1194576). EN is supported by an NHMRC (2021994) and Heart Foundation PhD Scholarship (106246) and a RACP Vincent Fairfax Family Foundation Research Entry Scholarship. The Hudson Institute of Medical Research is supported by the Victorian Government’s Operational Infrastructure Scheme. Funders had no role in study design, data collection, data analysis, data interpretation or manuscript writing.
