# Supplementary material for: Identifying unilateral aldosterone-producing adenomas using published algorithms and imaging: a systematic review and meta-analysis
Source: Endocr Connect. 2025 Nov 4;14(11):e250339. doi: 10.1530/EC-25-0339 (PMC12592847; doi:10.1530/EC-25-0339)
Supplement: Supplementary file 1 [file supplementary_materials.pdf]

Supplemental Materials

Table S1: Database search terms

Database	Medline (Ovid)		Embase (Ovid)	
Defined headings	"Primary aldosteronism"	"Outcome"	"Primary aldosteronism"	"Outcome"
Search terms	Terms combined with OR:<br><br>Hyperaldosteronism/<br>Hyperaldosteronism<br>Aldosteronism<br>Hyperaldosteronaemia<br>Hyperaldosteronemia<br>Hyper-aldosteronaemia<br>Hyper-aldosteronemia<br>Hyper-aldosteronism<br>Aldosteronaemia<br>Aldosteronemia<br>Conn's syndrome<br>Conn Syndrome<br>Conn* syndrome<br>Conn's disease<br>Conn disease<br>Conn* disease	Terms combined with OR:<br><br>Unilateral<br>Bilateral<br>Lateralis*<br>Lateraliz*<br>Localis*<br>Localiz*<br>Subtyp*	Terms combined with OR:<br><br>Primary Aldosteronism/<br>Hyperaldosteronism/<br>Hyperaldosteronism<br>Aldosteronism<br>Hyperaldosteronaemia<br>Hyperaldosteronemia<br>Hyper-aldosteronaemia<br>Hyper-aldosteronemia<br>Hyper-aldosteronism<br>Aldosteronaemia<br>Aldosteronemia<br>Conn* syndrome<br>Conn* disease	Terms combined with OR:<br><br>Unilateral<br>Bilateral<br>Lateralis*<br>Lateraliz*<br>Localis*<br>Localiz*<br>Subtyp*
Search run	[Primary aldosteronism] AND [Outcome]		[Primary aldosteronism] AND [Outcome]	
Limits	English only		English only	

*truncation wildcard used to search all words with the same preceding letters

Table S2: Characteristics of study cohorts

Characteristics	N = 49
Number of people with primary aldosteronism (PA)	66 (41, 147)
Number of people with unilateral PA that score was applied to	42 (25, 83)
Number of people with bilateral PA that score was applied to	24 (13, 63)
Country of recruitment	
Asia (excluding China)	9 (18%)
Canada	2 (4.1%)
China	12 (24%)
Europe	15 (31%)
New Zealand	1 (2.0%)
UK	5 (10%)
USA	5 (10%)
Study sites	
Single	36 (73%)
Multi-centre	13 (27%)
Screening method for PA	
Aldosterone-to-renin ratio (ARR)	19 (39%)
ARR and plasma aldosterone concentration (PAC)	12 (24%)
ARR and plasma renin activity (PRA)	2 (4.1%)
PRA	1 (2%)
Not specified	8 (16%)
Not specified beyond stating Endocrine Society guidelines were used	5 (10%)
Laboratory method of measuring aldosterone	
Immunoassay	28 (57%)
LCMS/MS	1 (2%)
Mix	1 (2%)
Not stated	19 (39%)
Type of renin measured	
Direct renin concentration (DRC)	6 (12%)
PRA	30 (61%)
Not stated	13 (27%)
Laboratory method of measuring renin	
Immunoassay	26 (53%)
LCMS/MS	2 (4%)
Not stated	21 (43%)
Confirmatory test used	
Saline suppression test (seated)	2 (4%)
Saline suppression test (recumbent)	6 (12%)
Saline suppression test (position not stated)	22 (45%)
Oral salt loading	6 (12%)
Fludrocortisone suppression test	3 (6%)
Captopril challenge test	17 (35%)
Frusemide upright test	4 (8%)
Not stated	12 (24%)
Multiple confirmatory tests used within cohort	17 (35%)
Interfering medications withheld	
Yes	33 (67%)

No	1 (2%)
Not stated	15 (31%)
Age of group with PA when provided as mean (n=19)	51.0 (49.4, 52.4)
Age of group with PA when provided as median (n=14)	51.0 (49.0, 52.0)
Age of group with bilateral PA when provided as mean (n=17)	51.6 (49.1, 52.2)
Age of group with bilateral PA when provided as median (n=15)	51.0 (48.5, 52.3)
Age of group with unilateral PA when provided as mean (n=17)	48.9 (47.4, 50.4)
Age of group with unilateral PA when provided as median (n=15)	50.0 (47.5, 51.0)
SBP when provided as mean (n=12)	154 (152, 164)
SBP when provided as median (n=8)	146 (142, 150)
SBP of group with bilateral PA when provided as mean (n=13)	154 (147, 168)
SBP of group with bilateral PA when provided as median (n=14)	148 (144, 154)
SBP of group with unilateral PA when provided as mean (n=13)	156 (149, 177)
SBP of group with unilateral PA when provided as median (n=14)	148 (143, 159)
DBP when provided as mean (n=11)	94 (92, 99)
DBP when provided as median (n=9)	90 (87, 92)
DBP of group with bilateral PA when provided as mean (n=12)	96 (91, 99)
DBP of group with bilateral PA when provided as median (n=15)	91 (90, 96)
DBP of group with unilateral PA when provided as mean (n=12)	92 (90, 104)
DBP of group with unilateral PA when provided as median (n=15)	91 (89, 98)
BMI when provided as mean (n=7)	25.4 (25.1, 26.6)
BMI when provided as median (n=8)	26.4 (25.1, 28.4)
Sex distribution (n=46)	
Number of males with PA	50 (21, 101)
Percentage of males with PA in study	57 (48, 65)
Number of females with PA	37 (20, 61)
Percentage of females with PA in study	43 (35, 52)
Serum potassium when provided as mean for overall group (n=11)	3.2 (2.9, 3.4)
Unilateral PA (n=17)	3.2 (3.0, 3.4)
Bilateral PA (n=17)	3.5 (3.4, 3.8)
Serum potassium when provided as median for overall group (n=6)	3.5 (3.4, 3.7)
Unilateral PA (n=14)	3.3 (3.0, 3.4)
Bilateral PA (n=13)	3.7 (3.6, 3.7)
AVS performed with ACTH stimulation	
Yes	22 (45%)
No	12 (24%)
Both (some with ACTH stimulation and some without)	7 (14%)
Not stated	8 (16%)
Sequential or simultaneous sampling during AVS	
Sequential	13 (27%)
Simultaneous	8 (16%)
Other	1 (2.1%)
Not stated	27 (55%)
Lateralisation criteria in the presence of ACTH use (n=29)	
Not applicable (ACTH stimulation not used)	21
2 of 3: 1) PAC > 1400 ng/dL at one side 2) LI > 4 3) Contralateral ratio < 1	1
LI > 2	1
LI > 2.6	1

LI > 3	2
LI > 4	16
LI > 4 and contralateral suppression	2
LI > 4 or LI 3-4 with contralateral suppression	1
LI > 5	2
LI ≥ 4.0 or 2.6 < LI < 4.0 with contralateral index < 1	1
Not specified	1
Lateralisation criteria in the absence of ACTH use (n=27)	
Not applicable (ACTH stimulation used)	22
LI > 2	7
LI > 3	1
LI > 4	12
LI > 4 or LI 3-4 with contralateral suppression	1
LI > 4 or LI 2-4 with contralateral suppression	1
Not specified	5
Criteria to determine successful cannulation in presence of ACTH (n=28)	
Not applicable (ACTH stimulation not used)	21
Cortisol level > 200 µg/dL in adrenal vein after ACTH stimulation	2
Digital subtraction angiography and retrograde CT adrenal venography after intravenous injection of contrast medium	1
SI > 2	2
SI > 3	8
SI > 5	13
Not specified	2
Criteria to determine successful cannulation in absence of ACTH (n=27)	
Not applicable (ACTH stimulation used)	22
Increase in either aldosterone or cortisol compared to baseline inferior vena cava values	1
SI > 2	13
SI > 3	7
Not specified	6

Data presented as Median (IQR) or n (%)
Abbreviations – ACTH: adrenocorticotropic hormone; ARR: aldosterone-to-renin ratio; AVS: adrenal vein sampling; BMI: body mass index; CT: computed tomography; DBP: diastolic blood pressure; DRC: direct renin concentration; LCMS/MS: liquid chromatography mass spectrometry; LI: lateralisation index; PA: primary aldosteronism; PAC: plasma aldosterone concentration; PRA: plasma renin activity; SBP: systolic blood pressure; SI: selectivity index

Table S3: Algorithms included in systematic review and reported diagnostic accuracy*

Algorithm	Surname of lead author (Year, Country)	Score variation or cohort if more than one in study	Subtype based on AVS (unilateral/ bilateral)	True positive	False positive	False negative	True negative	Sensitivity (%)	Specificity (%)	PPV (%)	NPV (%)
**Algorithms combining biochemical, radiological and demographic characteristics**											
Kupers' clinical prediction score<br>1) Typical Conn's adenoma on imaging** (3 points)<br>2) Hypokalaemia with serum potassium < 3.5 mmol/L (2 points)<br>3) eGFR (MDRD) < 80 mL/min/1.73sqm (0 points), eGFR 80-99 (1 point), eGFR ≥ 100 (2 points)	Kupers(2012, France)[1]	Score ≥ 5	49 / 38	26	0	23	37	53	100	100	62
	Burrello(2020, Italy and Germany)[2]	Score ≥ 5<br>Adrenal nodule ≥ 10 mm	133 / 82	82	19	51	63	62	77	81	55
	Zhang(2017, China)[3]	Score ≥ 5	211 / 195	67	35	144	160	32	82	66	53
	Riester(2014, Germany)[4^]	Score ≥ 5<br>Adrenal nodule ≥ 10 mm	127 / 66	58	13	69	53	46	80	82	43
	Riester(2014, Germany)[4]	Score ≥ 5<br>Adrenal nodule ≥ 10 mm<br>With LI reduced to 3 (from 4)	127 / 66	57	12	70	54	45	81	83	44
	Riester(2014, Germany)[4]	Score ≥ 5 with adrenal nodule ≥ 8 mm rather than 10 mm	127 / 66	62	15	65	51	49	77	81	44
	Venos(2014, Canada)[5]	Score ≥ 5	64 / 46	41	7	23	39	64	85	85	63
	Puar(2020, Singapore)[6]	Score ≥ 5	70 / 33	60	15	10	18	86	55	80	64
	Sze(2014, United Kingdom)[7]	Score ≥ 5	49 / 26	19	3	30	23	39	88	86	43
	Sam(2022, Canada)[8]	Score ≥ 5	157 / 106	95	26	62	80	61	75	79	56
	Song(2022, China)[9]	Score ≥ 5 using unilateral nodule ≥ 8 mm and serum potassium < 3.5 mmol/L<br>Development cohort	268 / 88	204	15	64	73	76	83	93	53
	Song(2022, China)[9]	Score ≥ 5 using unilateral nodule ≥ 8mm and serum potassium < 3.5 mmol/L<br>Validation cohort	84 / 117	22	2	62	115	26	98	92	65
	Song(2022, China)[9]	Score ≥ 5 using unilateral nodule ≥ 8mm and eGFR ≥ 100	268 / 88	83	12	185	76	31	86	87	29

Algorithm	Surname of lead author (Year, Country)	Score variation or cohort if more than one in study	Subtype based on AVS (unilateral/ bilateral)	True positive	False positive	False negative	True negative	Sensitivity (%)	Specificity (%)	PPV (%)	NPV (%)
		Development cohort									
	Song(2022, China)[9]	Score ≥ 5 using unilateral nodule ≥ 8mm and eGFR ≥ 100<br>Validation cohort	84 / 117	0	0	84	117		100		58
	Song(2022, China)[9]	Score using unilateral nodule ≥ 8mm, serum potassium < 3.5 mmol/L and eGFR ≥ 100<br>Development	268 / 88	72	6	196	82	27	93	92	29
	Song(2022, China)[9]	Score using unilateral nodule ≥ 8mm, serum potassium < 3.5 mmol/L and eGFR ≥ 100<br>Validation cohort	84 / 117	0	0	84	117		100		58
	Kolosova(2022, Czech Republic)[10]	Score using unilateral nodule ≥ 8mm, serum potassium < 3.5 mmol/L and eGFR ≥ 100<br>Development	96 / 54	49	6	47	48	51	89	89	51
	Sze(2014, United Kingdom)[7]	Score ≥ 5 using a radiological grading system score of 4 and 5[#]	49 / 26	32	5	17	21	65	81	86	55
	Riester(2014, Germany)[4]	Score ≥ 6	127 / 66	34	10	93	56	**27**	**85**	77	38
	Venos(2014, Canada)[5]	Score ≥ 6	64 / 46	25	2	39	44	39	96	93	53
	Riester(2014, Germany)[4]	Score ≥ 7	130 / 64	17	7	110	59	13	89	71	34
	Zhang(2017, China)[3]	Score of 4	211 / 195	131	92	80	103	62	53	59	56
Kupers' clinical prediction score with additional score band for potassium levels<br>1) Typical Conn's adenoma on imaging* (3 points)<br>2) Hypokalaemia with serum potassium <	Sze(2014, United Kingdom)[7]	Score ≥ 5 using a radiological grading system score of 5[#]	49 / 26	20	3	29	23	41	88	87	44
	Sze(2014, United Kingdom)[7]	Score ≥ 5 using a radiological grading system	49 / 26	33	5	16	21	67	81	87	57

Algorithm	Surname of lead author (Year, Country)	Score variation or cohort if more than one in study	Subtype based on AVS (unilateral/ bilateral)	True positive	False positive	False negative	True negative	Sensitivity (%)	Specificity (%)	PPV (%)	NPV (%)
3.5 mmol/L (2 points); 3.5-3.8 mmol/L (1 point), >3.8 mmol/L (0 points)<br>3) eGFR (MDRD) < 80 mL/min/1.73sqm (0 points), eGFR 80-99 (1 point), eGFR ≥ 100 (2 points)		score of 4 or 5#									
	Sam(2022, Canada)[8]	Score ≥ 5	157 / 106	99	27	58	79	63	75	79	58
Clinical radiomic model combining 5 selected variables (age, sex, serum potassium, Radscore, and ARR)	He(2021, China)[11]	Cut-off of 0.714<br>Training cohort	22 / 40	22	11	1	29	96	73	67	97
	He(2021, China)[11]	Cut-off of 0.548<br>Validation cohort	7 / 21	6	2	1	19	86	90	75	95
Both of:<br>1) PAC at 0600 ≥ 217.5 pg/mL<br>2) Unilateral adrenal nodule on CT	Kobayashi(2016, Japan)[12]		32 / 22	24	0	6	18	80	100	100	75
Both of:<br>1) Positive bedside test result<br>2) Adrenal nodule on CT<br>Bedside testing = plasma cortisol, PAC, PRA,18-hydroxycorticosterone (18-OH-B), and serum electrolytes obtained on the morning of study during supine bed rest and again after 2 hours of ambulation.<br>Positive test = fall in hormone levels with ambulation for 18-OH-B or PAC.	Phillips(2000, USA)[13]		41 / 8	12	0	29	8	29	100	100	22
All of:<br>1) Hypokalaemia with serum potassium < 3.6 mEq/L<br>2) Unilateral adrenal nodule on CT ≥ 5 mm<br>3) Age ≤ 45 years	Rossi(2022, Italy)[14]		128 / 100	68	0	60	100	53	100	100	63
	Song(2022, China)[9]	Development cohort	268 / 88	91	6	177	82	34	93	94	32
	Song(2022, China)[9]	Validation cohort	84 / 117	4	1	80	116	5	99	80	59
	Kolosova(2022, Czech Republic)[10]		96 / 54	13	1	83	53	14	98	93	39
Endocrine Society criteria, all of:<br>1) Spontaneous hypokalemia<br>2) PAC > 30 ng/dL (831 pmol/L)<br>3) Unilateral nodule > 10 mm and normal-appearing contralateral adrenal<br>4) 4) Age < 35 years	Sam(2022, Canada)[8]		152 / 119	4	0	148	119	3	100	100	45
	Song(2022, China)[9]	Development cohort	268 / 88	19	0	249	88	7	100	100	26
	Song(2022, China)[9]	Validation cohort	84 / 117	1	0	83	117	1	100	100	59
Modified Kupers' prediction score	Zhang(2017, China)[3]	Score ≥ 5	64 / 84	29	8	35	76	45	90	78	68

Algorithm	Surname of lead author (Year, Country)	Score variation or cohort if more than one in study	Subtype based on AVS (unilateral/ bilateral)	True positive	False positive	False negative	True negative	Sensitivity (%)	Specificity (%)	PPV (%)	NPV (%)
1) Typical Conn's adenoma on imaging* (2 points)<br>2) Hypokalaemia with serum potassium < 3.5 mmol/L (2 points)<br>3) Urinary aldosterone level (µg/24 hours): <13 (0 points), 13-19 (1 point), 19-23 (2 points), >23 (3 points)	Song(2022, China)[9]	Score ≥ 5<br>Development cohort	268 / 88	94	2	174	86	35	98	98	33
	Zhang(2017, China)[3]	Score ≥ 6	64 / 84	7	2	57	82	11	98	78	59
Age < 40 years <u>and</u> Modified Kupers' prediction score:<br>1) Typical Conn's adenoma on imaging* (2 points)<br>2) Hypokalaemia with serum potassium < 3.5 mmol/L (2 points)<br>3) Urinary aldosterone level (µg/24 hours): <13 (0 points), 13-19 (1 point), 19-23 (2 points), >23 (3 points)	Zhang(2017, China)[3]	Score ≥ 6	64 / 84	4	0	13	13	24	100	100	50
	Song(2022, China)[9]	Score ≥ 6<br>Development cohort	268 / 88	13	0	255	88	5	100	100	26
	Zhang(2017, China)[3]	Score ≥ 5	64 / 84	13	2	4	11	76	85	87	73
All 4 of:<br>1) Serum potassium ≤ 3.5 mmol/L<br>2) PAC ≥ 20 ng/dL (554 pmol/L)<br>3) PRC ≤ 5 mIU/mL<br>4) Unilateral adrenal nodule ≥ 10 mm on CT	Song(2022, China)[9]	Development cohort	268 / 88	140	0	128	88	52	100	100	41
	Song(2022, China)[9]	Validation cohort	84 / 117	13	0	71	117	15	100	100	62
	Kolosova(2022, Czech Republic)[10]	Validation cohort	94 / 44	33	5	61	39	35	89	87	39
Age ≤ 40 years and unilateral adenoma on CT ≥ 10 mm	Kupers(2012, France)[1]	France	49 / 38	9	0	40	38	18	100	100	49
	Song(2022, China)[9]	Development cohort	268 / 88	56	5	212	83	21	94	92	28
	Song(2022, China)[9]	Validation cohort	84 / 117	5	4	79	114	6	97	56	59
	Kolosova(2022, Czech Republic)[10]	Development cohort	96 / 54	12	0	84	54	13	100	100	39
Age ≤ 45 years and unilateral adenoma on CT ≥ 10 mm	Rossi(2022, Italy)[14]	Italy	128 / 100	93	5	35	95	73	95	95	73
Age < 40 years <u>and</u> Kupers' score ≥ 5<br>1) Typical Conn's adenoma on imaging* (3 points)<br>2) Hypokalaemia with serum potassium < 3.5 mmol/L (2 points)<br>3) eGFR (MDRD) < 80 mL/min/1.73sqm	Riester(2014, Germany)[4]	Germany	22 / 6	13	0	9	6	59	100	100	40

Algorithm	Surname of lead author (Year, Country)	Score variation or cohort if more than one in study	Subtype based on AVS (unilateral/ bilateral)	True positive	False positive	False negative	True negative	Sensitivity (%)	Specificity (%)	PPV (%)	NPV (%)
(0 points), eGFR 80-99 (1 point), eGFR ≥ 100 (2 points)											
All of:<br>1) Hypokalaemia (serum potassium < 3.5 mEq/L)<br>2) PAC > 30 ng/dL (831 pmol/L)<br>3) Unilateral lesion on CT ≥ 7 mm	Lee(2021, Korea)[15]	Korea	309 / 157	125	14	184	143	40	91	90	44
	Song(2022, China)[9]	Development cohort	268 / 88	131	4	137	84	49	95	97	38
	Kolosova(2022, Czech Republic)[10]	Development cohort	96 / 54	36	4	60	50	38	93	90	45
	Song(2022, China)[9]	Validation cohort	84 / 117	16	0	68	117	19	100	100	63
All of:<br>1) Age ≤ 40 years<br>2) Unilateral disease on CT (adrenal nodule > 10 mm diameter and normal contralateral adrenal gland)<br>3) PAC > 15.9 ng/dL (440 pmol/L)<br>4) Serum potassium < 3.5 mEq/L	Umakoshi(2018, Japan)[16]		262 / 96	58	11	204	85	22	89	84	30
All of:<br>1) Age ≤ 35 years<br>2) Unilateral disease on CT (adrenal nodule > 10 mm diameter and normal contralateral adrenal gland)<br>3) PAC > 15.9 ng/dL (440 pmol/L)<br>4) Serum potassium < 3.5 mEq/L	Umakoshi(2018, Japan)[16]		262 / 96	27	3	235	93	10	97	90	28
	Song(2022, China)[9]	Development cohort	268 / 88	32	2	236	86	12	98	94	27
	Kolosova(2022, Czech Republic)[10]	Development cohort	96 / 54	4	0	92	54	4	100	100	37
	Song(2022, China)[9]	Validation cohort	84 / 117	1	0	83	117	1	100	100	59
All of:<br>1) Age 35 - 40 years<br>2) Unilateral disease on CT (adrenal nodule > 10 mm diameter and normal contralateral adrenal gland)<br>3) PAC > 15.9 ng/dL (440 pmol/L)<br>4) Serum potassium < 3.5 mEq/L	Umakoshi(2018, Japan)[16]		262 / 96	31	8	231	88	12	92	80	28
All of:<br>1) Age > 40 years<br>2) Unilateral disease on CT (adrenal nodule > 10 mm diameter and normal contralateral adrenal	Umakoshi(2018, Japan)[16]		262 / 96	198	91	64	5	76	11	69	7

Algorithm	Surname of lead author (Year, Country)	Score variation or cohort if more than one in study	Subtype based on AVS (unilateral/ bilateral)	True positive	False positive	False negative	True negative	Sensitivity (%)	Specificity (%)	PPV (%)	NPV (%)
gland)<br>3) PAC > 15.9 ng/dL (440 pmol/L)<br>4) Serum potassium < 3.5 mEq/L											
**Algorithms involving confirmatory testing and ACTH stimulation testing**											
20-point SPACE score:<br>1) PAC at screening: > 25 ng/dL (693 pmol/L) (0.5 points)<br>2) Lowest potassium: < 3.4 mEq/L (5 points), 3.4-3.9 (1.5 points), ≥ 4 (0 points)<br>3) PAC post-confirmatory testing: ≤ 15 ng/dL (416 pmol/L) (0 points), 15.1-19.9 (416-551 pmol/L) (1 point), ≥ 20 ng/dL (554 pmol/L) (2 points)<br>4) Nodule at CT scanning (4 points)<br>5) Largest nodule at CT scanning: ≤ 10 mm (0 points), 11-30 mm (1 point), > 30 mm (2 points)<br>6) CT findings: bilaterally normal (0 points), bilaterally abnormal (4.5 points), unilateral abnormality (6.5 points)	Burrello(2020, Italy)[2]	Score > 12<br>Training cohort	93 / 57	87	10	6	47	94	79	88	88
	Burrello(2020, Italy)[2]	Score > 12<br>Validation cohort	40 / 25	35	7	5	18	88	72	83	78
	Kocjan(2022, Slovenia)[17]	Score > 12	59 / 85	41	30	18	55	69	65	58	75
	Burrello(2020, Italy)[2]	Score > 8<br>Combined training and validation cohort	133 / 82	129	54	4	28	97	34	70	88
	Burrello(2020, Italy)[2]	Score > 8<br>Training cohort	93 / 57	91	36	2	21	98	37	72	91
	Burrello(2020, Italy)[2]	Score > 8<br>Validation cohort	40 / 25	38	18	2	7	95	28	68	78
	Burrello(2020, Italy)[2]	Score > 16<br>Combined training and validation cohort	133 / 82	59	3	74	79	44	96	95	52
	Burrello(2020, Italy)[2]	Score > 16<br>Training cohort	93 / 57	44	1	49	56	47	98	98	53
	Burrello(2020, Italy)[2]	Score > 16<br>Validation cohort	40 / 25	15	2	25	23	38	92	88	48
	Burrello(2020, Italy)[2]	Score > 16<br>Validation cohort	57 / 61	50	18	7	43	88	70	74	86
	Kocjan(2022, Slovenia)[17]	Score > 16	59 / 85	25	7	34	78	42	92	78	70
Both of:<br>1) PAC post-captopril challenge test (CCT) > 21.2 ng/dL (588 pmol/L)<br>2) Unilateral adrenal nodule with a normal contralateral gland on CT	Chen(2021, China)[18]		71 / 47	54	2	17	45	76	96	96	73
Both of:<br>1) PAC post-saline suppression test	Chen(2021, China)[18]		71 / 47	61	1	10	46	86	98	98	82

Algorithm	Surname of lead author (Year, Country)	Score variation or cohort if more than one in study	Subtype based on AVS (unilateral/ bilateral)	True positive	False positive	False negative	True negative	Sensitivity (%)	Specificity (%)	PPV (%)	NPV (%)
(SST) > 17.2 ng/dL (477 pmol/L)<br>2) Unilateral adrenal nodule with a normal contralateral gland on CT											
Both of:<br>1) Fall in PAC after a 4 hour postural stimulation test (supine overnight, PAC measured at 0800 and after 4 hours of continued erect posture)<br>2) Adrenal nodule on CT	Espiner(2003, New Zealand)[19]		26 / 7	10	0	16	7	38	100	100	35
1) Unilateral adrenal nodule ≥ 6 mm (2 points)<br>2) PAC after SST ≥ 165 ng/L (457 pmol/L) (1 point)	Holaj(2022, Czech Republic)[20]	Development cohort	96 / 54	46	0	50	54	48	100	100	52
	Kolosova(2022, Czech Republic)[10]	Validation cohort	94 / 44	34	0	60	44	36	100	100	42
Supervised machine learning – linear discriminant model (LDA) involving six selected variables:<br>1) PAC at screening<br>2) PAC after confirmatory testing<br>3) Lowest potassium recorded in the absence of diuretic therapy<br>4) Presence/absence of a nodule at CT scanning imaging<br>5) Nodule diameter<br>6) Descriptive CT scanning findings	Burrello(2020, Italy)[2]	Training cohort	93 / 57	115	22	18	60	86	73	84	77
Supervised machine learning – random forest (RF) model involving six selected variables:<br>1) PAC at screening<br>2) PAC after confirmatory testing<br>3) Lowest potassium recorded in the absence of diuretic therapy<br>4) Presence/absence of a nodule at CT scanning imaging<br>5) Nodule diameter<br>6) Descriptive CT scanning findings	Burrello(2020, Italy)[2]	Training cohort	93 / 57	132	14	1	68	99	83	90	99
	Song(2022, China)[9]	Unilateral nodule > 14mm Validation cohort	84 / 117	27	16	57	101	32	86	63	64
	Song(2022, China)[9]	Unilateral nodule > 14mm Development cohort	268 / 88	121	4	147	84	45	95	97	36
	Song(2022, China)[9]	Unilateral nodule ≥ 8 mm, lowest potassium ≤ 3.9 mmol/L, PAC post-CCT or SST > 8.9 ng/dL (247 pmol/L) and PAC at screening > 30.3 ng/dL (839 pmol/L)<br>Validation cohort	84 / 117	8	1	76	116	10	99	89	60

Algorithm	Surname of lead author (Year, Country)	Score variation or cohort if more than one in study	Subtype based on AVS (unilateral/ bilateral)	True positive	False positive	False negative	True negative	Sensitivity (%)	Specificity (%)	PPV (%)	NPV (%)
	Kolosova(2022, Czech Republic)[10]	Unilateral nodule ≥ 8 mm, lowest potassium ≤ 3.9 mmol/L, PAC post-CCT or SST > 8.9 ng/dL (247 pmol/L) and PAC at screening > 30.3 ng/dL (839 pmol/L)	96 / 54	38	0	58	54	40	100	100	48
	Song(2022, China)[9]	Unilateral nodule ≥ 8 mm, lowest potassium ≤ 3.9 mmol/L, PAC post-CCT or SST > 8.9 ng/dL (247 pmol/L) and PAC at screening > 30.3 ng/dL (839 pmol/L)<br>Development cohort	268 / 88	123	1	145	87	46	99	99	38
All of:<br>1) Unilateral adrenal lesion on CT<br>2) Serum potassium < 3.4 mEq/L<br>3) PAC ratio < 1.45 after frusemide upright posture test<br>4) Age < 52 years	Minami(2008, Japan)[21]		12 / 8	6	0	6	8	50	100	100	57
Both of:<br>1) PAC > 77.90 ng/dL (2161 pmol/L) 120 minutes after ACTH (IV 50 IU at 0800 after 1 mg dexamethasone at midnight)<br>2) Unilateral CT findings	Jiang(2015, China)[22]		42 / 13	41	2	15	37	73	95	95	71
**Anatomical imaging**											
CT imaging	Doppman(1992, USA)[23]		21 / 3	16	2	6	1	73	33	89	14
	Ladurner(2017, Germany)[24]		80 / 2	57	1	23	1	71	50	98	4
	Lee(2021, Korea)[15]		309 / 157	248	105	61	52	80	33	70	46
	Magill(2001, USA)[25]		15 / 21	8	8	7	13	53	62	50	65
	Sarlon-Bartoli(2011,		35 / 23	29	16	0	13	100	45	64	100

Algorithm	Surname of lead author (Year, Country)	Score variation or cohort if more than one in study	Subtype based on AVS (unilateral/ bilateral)	True positive	False positive	False negative	True negative	Sensitivity (%)	Specificity (%)	PPV (%)	NPV (%)
	France)[26]										
	Hu(2023, China)[27]		43 / 57	35	37	8	20	81	35	49	71
	Lau(2012, UK)[28]		29 / 14	10	1	19	13	34	93	91	41
	Raman(2015, USA)[29]		40 / 16	23	12	17	4	58	25	66	19
	Yen(2009, Taiwan)[30]		18 / 9	18	7	4	2	82	22	72	33
	Kim(2024)[31]		157 / 107	138	69	19	38	88	36	67	67
Anatomical imaging (combined MRI and CT imaging)	Gkaniatsa(2021, Sweden)[32]		25 / 20	20	5	4	12	83	71	80	75
MRI	Ladurner(2017, Germany)[24]		81 / 6	57	0	24	6	70	100	100	20
	Sohaib(2000, UK)[33]		10 / 10	7	0	3	10	70	100	100	77
Left-to-right adrenal volume ratio (L/Rv) on CT imaging after contrast administration using 1 mm slice thickness	Li(2019, China)[34]	Cut-off of L/Rv > 1.344 for predicting left PA	50 / 64	40	14	10	50	80	78	74	83
	Li(2019, China)[34]	Cut-off of L/Rv > 1.908 for predicting left-sided PA	50 / 64	23	0	27	64	46	100	100	70
	Li(2019, China)[34]	Cut-off of L/Rv < 1.267 for predicting right-sided PA	33 / 81	29	22	4	59	88	73	57	94
	Li(2019, China)[34]	Cut-off of L/Rv < 0.765 for predicting right-sided PA	33 / 31	9	0	24	81	27	100	100	77
	Li(2019, China)[34]	Cut-off of L/Rv > 1.908 for predicting left-sided PA for age < 35 years	7 / 6	5	0	2	6	71	100	100	75
	Li(2019, China)[34]	Cut-off of L/Rv > 1.908 for predicting left-sided PA for age ≥ 35 years	43 / 58	18	0	25	58	42	100	100	70
	Li(2019, China)[34]	Cut-off of L/Rv < 0.765 for predicting right-sided PA for age < 35 years	2 / 11	1	0	1	11	50	100	100	92
	Li(2019, China)[34]	Cut-off of L/Rv < 0.765 for predicting right-sided PA for age ≥ 35 years	31 / 70	8	0	23	70	26	100	100	75

Algorithm	Surname of lead author (Year, Country)	Score variation or cohort if more than one in study	Subtype based on AVS (unilateral/ bilateral)	True positiv e	Fals e posi tive	Fals e nega tive	True nega tive	Sen sitivi ty (%)	Spe cifici ty (%)	PPV (%)	NPV (%)
	Li(2019, China)[34]	Cut-off of L/Rv > 1.908 for predicting left-sided PA OR L/Rv < 0.765 for predicting right-sided PA	83 / 31	32	0	51	31	39	100	100	38
Left-versus-right-adrenal-volume ratio (L/Rv) and left-subtract-right-adrenal-volume difference (L-Rv)	Zhang(2023, China)[35]	L/Rv > 1.431 and L-Rv > 3.185 - to predict left-sided PA	46 / 72	12	2	34	70	26	97	86	67
	Zhang(2023, China)[35]	L/RV < 0.892 and L-Rv < -0.640 - to predict right sided PA	35 / 72	17	3	18	69	49	96	85	79
**Functional imaging**											
11C-Metomidate PET-CT imaging<br>- Dexamethasone pre-treatment 0.5 mg four times a day for 72 hours before the scan	Burton(2012, UK)[36]	LI (SUV ratio of tumour over normal adrenal) > 1.25	25 / 10	19	2	6	13	76	87	90	68
	Puar(2022, Singapore)[37]	LI > 1.25 (SUVmax in the nodule more than 1.25 times compared with the contralateral adrenal gland)	22 / 3	18	1	4	2	82	67	95	33
	Wu(2022, UK)[38]	LI (SUVmax ratio of tumor to normal background) >1.25 (using outcome of complete biochemical success)	69 / 47	51	6	18	41	74	87	89	69
	Wu(2022, UK)[38]	LI (SUVmax ratio of tumor to normal background) >1.25 (using outcome of complete or partial biochemical success)	74 / 42	55	2	19	40	74	95	97	67
	Burton(2012, UK)[36]	LI > 1.25 and tumor SUVmax > 17	25 / 10	16	0	9	13	64	100	100	59
	Soinio(2020, Finland)[39]	Metabolic activity localized to an anatomic adenoma and/ or >15% difference in SUVmax values between the adrenal glands. Optimal cut-off point of 1.16:1	34 / 21	11	5	9	4	55	44	69	31
68Ga-Pentixafor PET/CT	Ding(2020, China)[40]	Visual analysis	25 / 4	25	3	0	11	100	79	89	100

Algorithm	Surname of lead author (Year, Country)	Score variation or cohort if more than one in study	Subtype based on AVS (unilateral/ bilateral)	True positive	False positive	False negative	True negative	Sensitivity (%)	Specificity (%)	PPV (%)	NPV (%)
	Ding(2020, China)[40]	Lesion-to-liver ratio ≥ 2.36	25 / 4	25	0	0	14	100	100	100	100
	Ding(2020, China)[40]	Maximum standardized uptake value (SUVmax) of the adrenal lesion ≥ 11.18	25 / 4	22	0	3	14	88	100	100	82
	Ding(2020, China)[40]	LI ≥ 2.12	25 / 4	25	1	0	13	100	93	96	100
	Hu(2023, China)[27]	LI > 1.65 at 10 minutes with a unilateral adrenal nodule ≥ 10 mm on CT	43 / 57	33	0	10	57	77	100	100	85
	Hu(2023, China)[27]	LI > 1.57 at 40 minutes with a unilateral adrenal nodule ≥ 10 mm on CT	43 / 57	37	5	6	52	86	91	88	90
	Hu(2023, China)[27]	LI > 3.15 at 40 minutes with a unilateral adrenal nodule ≥ 10 mm on CT	43 / 57	19	0	24	57	44	100	100	70
	Yin(2024, China)[41]	LI > 1.39	19 / 7	17	0	2	7	90	100	100	78
	Yin(2024, China)[41]	SUVmax > 5.71	19 / 7	15	0	4	7	79	100	100	64
NP-59 imaging<br>- Dexamethasone 4 mg in divided doses for 7 days, Lugol's iodine for 24 hours before NP-59 injection (1 mCi) and during imaging intervals.	Gross(1985, USA)[42]	Adrenal images 5 days after NP59	30 / 20	22	17	8	3	73	15	56	27
	Gross(1985, USA)[42]	Adrenal images 3 or 4 days after NP59	30 / 20	30	0	0	20	100	100	100	100
NP-59 imaging<br>- Dexamethasone 2 mg in divided doses and potassium iodide (50 mg/day) for 3 days before and 7 days after NP-59 injection (37 MBq, 1 mCi)<br>- Images taken 7 days after tracer injection	Saiga(2020, Japan)[43]	Lateralisation index 2 (LI2) for NP59 defined in study as maximum count on the high value side divided by the low value<br><br>LI2 threshold 2.55 to detect microtumours or macrotumours	12 / 17	4	0	8	17	33	100	100	68
	Saiga(2020, Japan)[43]	LI2 threshold 2.55 + CT tumour > 1cm to detect macrotumours	9 / 5	4	0	5	5	44	100	100	50

Algorithm	Surname of lead author (Year, Country)	Score variation or cohort if more than one in study	Subtype based on AVS (unilateral/ bilateral)	True positive	False positive	False negative	True negative	Sensitivity (%)	Specificity (%)	PPV (%)	NPV (%)
	Saiga(2020, Japan)[43]	LI2 threshold 1.80 to detect microtumours or macrotumours	12 / 17	7	1	5	16	58	94	88	76
NP-59 imaging<br>- Dexamethasone 8 mg daily and Lugol's solution 1 mL daily for 3 days before and 5 days after the NP-59 injection (37 MBq, 1mCi)<br>- Images taken 72, 96 and 120 hours after tracer injection	Yen(2009, Taiwan)[30]	Planar imaging	18 / 9	9	3	13	6	41	67	75	32
	Yen(2009, Taiwan)[30]	SPECT/CT	18 / 9	18	3	4	6	82	67	86	60
	Yen(2009, Taiwan)[30]	SPECT	18 / 9	15	3	7	6	68	67	83	46

Abbreviations: ACTH: adrenocorticotropic hormone; ARR: aldosterone-to-renin ratio; CT: computed tomography; eGFR: estimated glomerular filtration rate; IU: international unit; IV: intravenous; LI: lateralisation index; MRI: magnetic resonance imaging; PA: primary aldosteronism; PAC: plasma aldosterone concentration; PRC: plasma renin concentration; SPECT: single-photon emission computerized tomography; SST: saline suppression test; SUVmax: maximum standardised uptake value

* Sensitivity, specificity, PPV and NPV were calculated based on applying values reported in publications reviewed to identify the number of cases in the categories of true positive, false positive, true negative and false negative.

**Kupers' score specified criteria for imaging: unilateral radiolucent nodule (<10 HU) with the remaining ipsilateral and contralateral glands appearing smooth and not enlarged.

^Calculated based on raw numbers and specificity reported in text, resulting in slight difference from values reported in table.[4]

Radiological Grading Score (RGS) by Sze et al: RGS 5 = definitely unilateral (definite unilateral nodule and contralateral adrenal gland has completely normal morphology with limb thickness ≤5 mm and body ≤1 cm); RGS 4 = probably unilateral (focal nodule with thickened limbs in the ipsilateral or contralateral gland)

Figure S1: Plot of sensitivity vs specificity for studies of algorithms combining biochemical, radiological and demographic characteristics

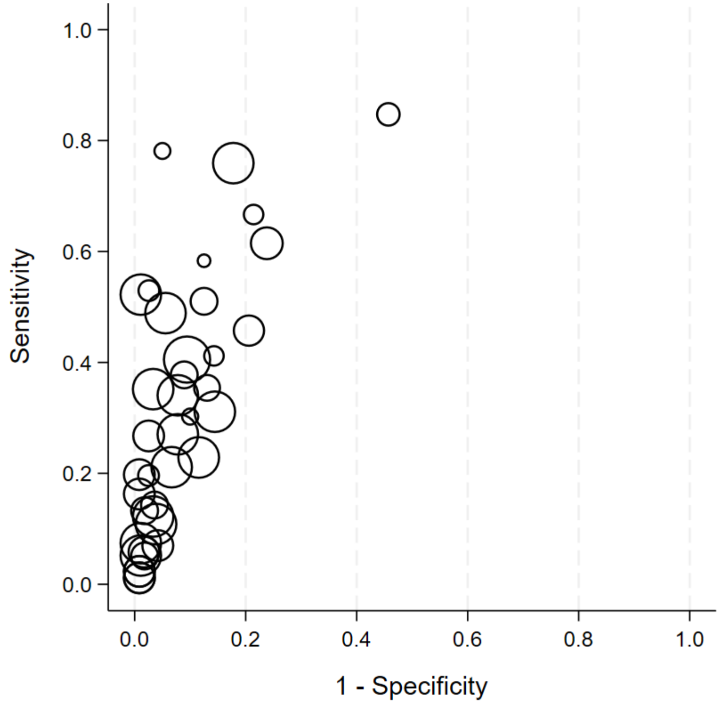

Figure S2: Plot of sensitivity vs specificity for studies of algorithms involving confirmatory testing

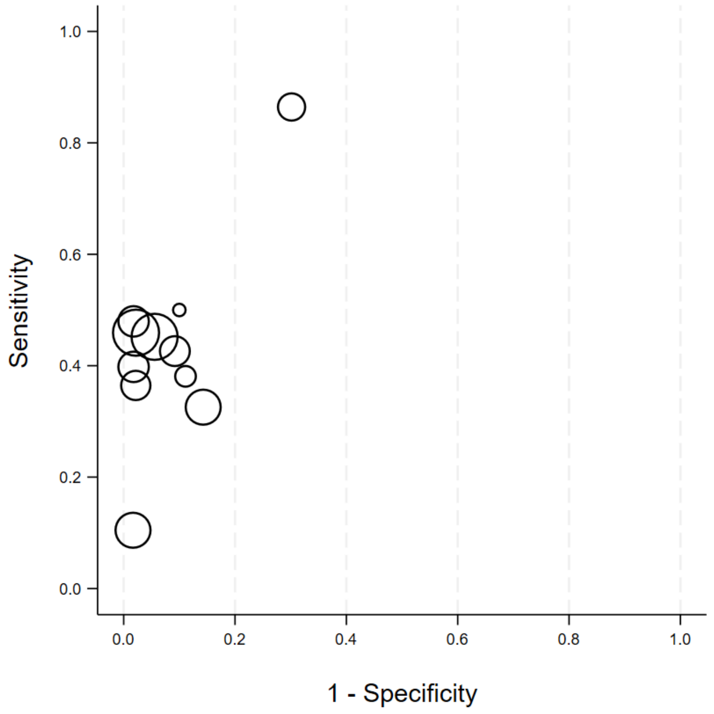

Figure S3: Plot of sensitivity vs specificity for studies involving anatomical imaging

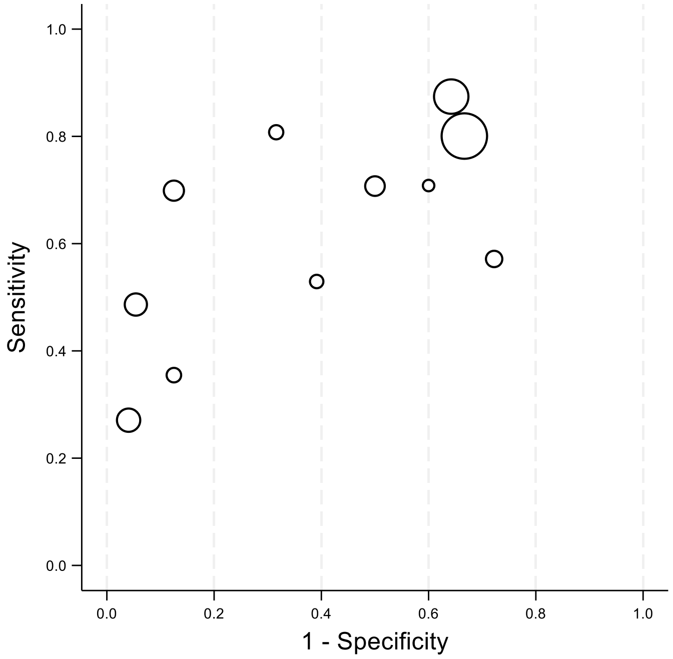

Figure S4: Deek's funnel plot to assess for publication bias

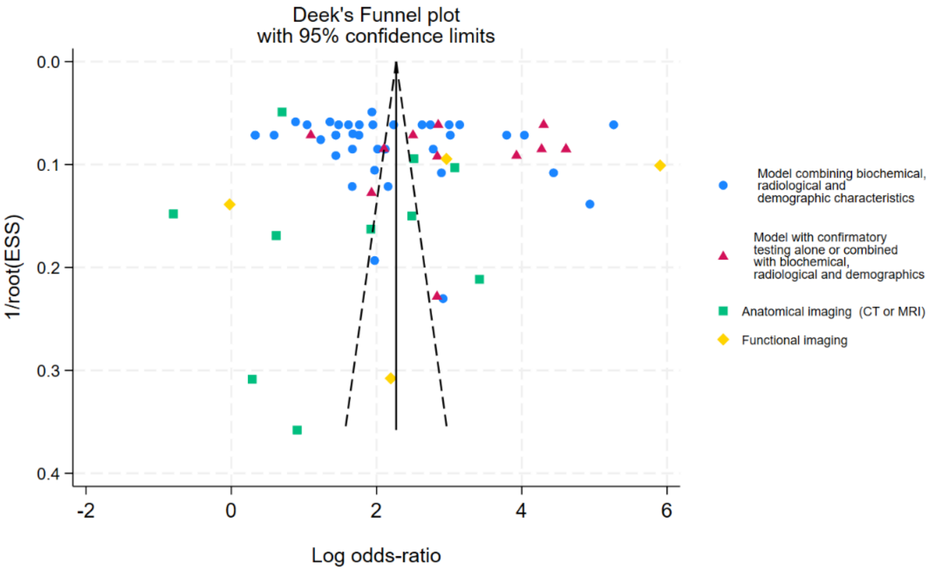

[Tests for publication bias; p=0.213 for Category 1, p=0.676 for Category 2 & p=0.543 for Category 5]

Figure S5: Risk of bias assessment of included studies based on QUADAS-2 criteria

5a) Summary plot of total risk of bias per category

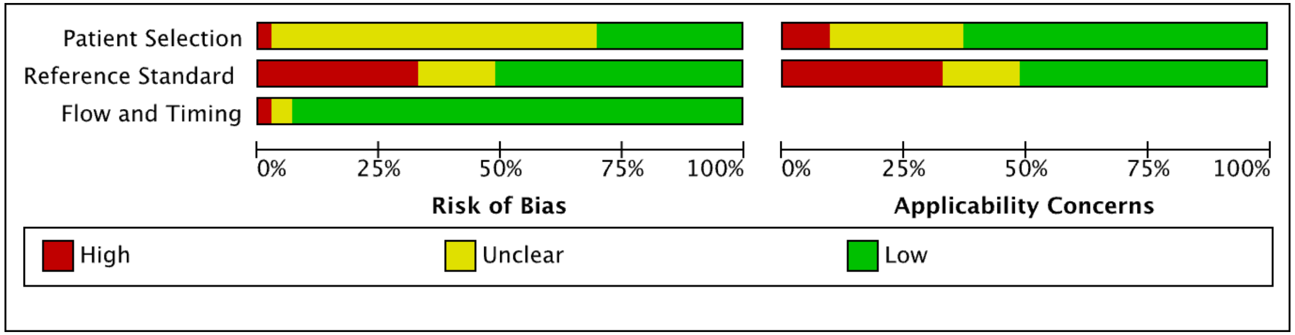

Approach to QUADAS grading

For patient selection, risk of bias was considered unclear if it was not explicitly stated that recruitment was consecutive or that all relevant patients in a specified duration were evaluated. Concerns regarding applicability was considered high if details were not provided of how the diagnosis of PA was made or biochemistry was not provided. Concerns regarding applicability was considered unclear where studies stated the diagnosis of PA was made in accordance with guidelines but detail was not provided, or the parameters used for screening were provided without further details.

For the reference standard (AVS), risk of bias was graded unclear if the details of AVS or its interpretation were not given, including if either no gradient for LI or SI was specified. Concerns regarding applicability were graded as high where 1) LI < 4 was used with or without ACTH use (LI 2-4 with contralateral suppression was accepted to be of low concern), 2) SI < 3 in the case of ACTH-stimulated AVS or SI < 2 for unstimulated AVS and 3) if a raw value was used to confirm successful cannulation rather than a selectivity index.

Figure S5b) Risk of bias per category in each individual study

	Risk of Bias: Patient Selection	Risk of Bias: Reference Standard	Risk of Bias: Flow and Timing	Applicability Concerns: Patient Selection	Applicability Concerns: Reference Standard
Burrello(2020)	+	+	+	+	+
Burton(2012)	?	?	+	?	?
Chen(2021)	?	−	+	+	−
Ding(2020)	?	?	?	?	?
Doppman(1992)	?	?	+	−	?
Espiner(2003)	?	−	+	?	−
Gkaniatsa(2021)	?	+	+	?	+
Gross(1985)	?	?	+	−	?
He(2021)	+	−	+	?	−
Holaj(2022)	?	+	+	+	+
Hu(2023)	?	+	+	+	+
Jiang(2015)	+	−	+	+	−
Kim(2024)	+	+	+	+	+
Kobayashi(2016)	?	−	+	+	−
Kocjan(2022)	?	+	+	+	+
Kolosova(2022)	?	+	+	+	+
Kupers(2012)	+	+	+	+	+
Ladurner(2017)	+	?	+	+	?
Lau(2012)	+	+	−	?	+
Lee(2021)	?	+	+	+	+
Li(2019)	?	−	+	+	−
Magill(2001)	?	+	+	?	+
Minami(2008)	?	−	+	+	−
Phillips(2000)	?	?	+	?	?
Puar(2020)	?	+	+	+	+
Puar(2022)	+	+	+	+	+
Raman(2015)	?	+	+	−	+
Riester(2014)	?	+	+	+	+
Rossi(2022)	?	−	?	?	−
Saiga(2020)	?	−	+	+	−
Sam(2022)	+	−	+	+	−
Sarlon-Bartoli(2011)	+	−	+	+	−
Sohaib(2000)	?	?	+	−	?
Soinio(2020)	+	+	+	?	+
Song(2022)	?	+	+	+	+
Sze(2014)	+	+	+	+	+
Umakoshi(2018)	?	+	+	+	+
Venos(2014)	?	−	+	+	−
Wu(2023)	?	+	+	+	+
Yen(2009)	?	−	+	?	−
Yin(2024)	+	+	+	+	+
Zhang(2017)	?	−	+	?	−
Zhang(2023)	−	+	+	+	+

− High   ? Unclear   + Low

References

1. Küpers EM, Amar L, Raynaud A, Plouin PF, Steichen O. A clinical prediction score to diagnose unilateral primary aldosteronism. J Clin Endocrinol Metab. 2012;97(10):3530-7.
2. Burrello J, Burrello A, Pieroni J, Sconfienza E, Forestiero V, Rabbia P, et al. Development and validation of prediction models for subtype diagnosis of patients with primary aldosteronism. J Clin Endocrinol Metab. 2020;105(10).
3. Zhang Y, Niu W, Zheng F, Zhang H, Zhou W, Shen Z, et al. Identifying unilateral disease in Chinese patients with primary aldosteronism by using a modified prediction score. J Hypertens. 2017;35(12):2486-92.
4. Riester A, Fischer E, Degenhart C, Reiser MF, Bidlingmaier M, Beuschlein F, et al. Age below 40 or a recently proposed clinical prediction score cannot bypass adrenal venous sampling in primary aldosteronism. J Clin Endocrinol Metab. 2014;99(6):E1035-9.
5. Venos ES, So B, Dias VC, Harvey A, Pasieka JL, Kline GA. A clinical prediction score for diagnosing unilateral primary aldosteronism may not be generalizable. BMC Endocr Disord. 2014;14:94.
6. Puar TH, Loh WJ, Lim DS, Loh LM, Zhang M, Foo RS, et al. Aldosterone-potassium ratio predicts primary aldosteronism subtype. J Hypertens. 2020;38(7):1375-83.
7. Sze WC, Soh LM, Lau JH, Reznek R, Sahdev A, Matson M, et al. Diagnosing unilateral primary aldosteronism - comparison of a clinical prediction score, computed tomography and adrenal venous sampling. Clin Endocrinol (Oxf). 2014;81(1):25-30.
8. Sam D, Kline GA, So B, Hundemer GL, Pasieka JL, Harvey A, et al. External validation of clinical prediction models in unilateral primary aldosteronism. Am J Hypertens. 2022;35(4):365-73.
9. Song Y, Yang J, Shen H, Ng E, Fuller PJ, Feng Z, et al. Development and validation of model for sparing adrenal venous sampling in diagnosing unilateral primary aldosteronism. J Hypertens. 2022;40(9):1692-701.
10. Kološová B, Waldauf P, Wichterle D, Kvasnička J, Zelinka T, Petrák O, et al. Validation of Existing Clinical Prediction Tools for Primary Aldosteronism Subtyping. Diagnostics. 2022;12(11):2806.
11. He K, Zhang ZT, Wang ZH, Wang Y, Wang YX, Zhang HZ, et al. A Clinical-Radiomic Nomogram Based on Unenhanced Computed Tomography for Predicting the Risk of Aldosterone-Producing Adenoma. Front Oncol. 2021;11:634879.
12. Kobayashi H, Haketa A, Ueno T, Suzuki R, Aoi N, Ikeda Y, et al. Subtype prediction in primary aldosteronism: measurement of circadian variation of adrenocortical hormones and 24-h urinary aldosterone. Clin Endocrinol (Oxf). 2016;84(6):814-21.
13. Phillips JL, Walther MM, Pezzullo JC, Rayford W, Choyke PL, Berman AA, et al. Predictive value of preoperative tests in discriminating bilateral adrenal hyperplasia from an aldosterone-producing adrenal adenoma. J Clin Endocrinol Metab. 2000;85(12):4526-33.
14. Rossi GP, Crimì F, Rossitto G, Amar L, Azizi M, Riester A, et al. Feasibility of imaging-guided adrenalectomy in young patients with primary aldosteronism. Hypertension. 2022;79(1):187-95.
15. Lee SH, Kim JW, Yoon HK, Koh JM, Shin CS, Kim SW, et al. Diagnostic accuracy of computed tomography in predicting primary aldosteronism subtype according to age. Endocrinol Metab (Seoul). 2021;36(2):401-12.
16. Umakoshi H, Ogasawara T, Takeda Y, Kurihara I, Itoh H, Katabami T, et al. Accuracy of adrenal computed tomography in predicting the unilateral subtype in young patients with hypokalaemia and elevation of aldosterone in primary aldosteronism. Clin Endocrinol (Oxf). 2018;88(5):645-51.
17. Kocjan T, Vidmar G, Popović P, Stanković M. Validation of three novel clinical prediction tools for primary aldosteronism subtyping. Endocr Connect. 2022;11(5).
18. Chen S, Liu H, Luo P, Yu Y. Computed tomography combined with confirmatory tests for the diagnosis of aldosterone-producing adenoma. Endocr J. 2021;68(3):299-306.
19. Espiner EA, Ross DG, Yandle TG, Richards AM, Hunt PJ. Predicting surgically remedial primary aldosteronism: role of adrenal scanning, posture testing, and adrenal vein sampling. J Clin Endocrinol Metab. 2003;88(8):3637-44.

20. Holaj R, Waldauf P, Wichterle D, Kvasnička J, Zelinka T, Petrák O, et al. Adrenal venous sampling could be omitted before surgery in patients with Conn's adenoma confirmed by computed tomography and higher normal aldosterone concentration after saline infusion test. Diagnostics (Basel). 2022;12(7):1718.
21. Minami I, Yoshimoto T, Hirono Y, Izumiyama H, Doi M, Hirata Y. Diagnostic accuracy of adrenal venous sampling in comparison with other parameters in primary aldosteronism. Endocr J. 2008;55(5):839-46.
22. Jiang Y, Zhang C, Wang W, Su T, Zhou W, Jiang L, et al. Diagnostic value of ACTH stimulation test in determining the subtypes of primary aldosteronism. J Clin Endocrinol Metab. 2015;100(5):1837-44.
23. Doppman JL, Gill JR, Jr., Miller DL, Chang R, Gupta R, Friedman TC, et al. Distinction between hyperaldosteronism due to bilateral hyperplasia and unilateral aldosteronoma: reliability of CT. Radiology. 1992;184(3):677-82.
24. Ladurner R, Sommerey S, Buechner S, Dietz A, Degenhart C, Hallfeldt K, et al. Accuracy of adrenal imaging and adrenal venous sampling in diagnosing unilateral primary aldosteronism. Eur J Clin Invest. 2017;47(5):372-7.
25. Magill SB, Raff H, Shaker JL, Brickner RC, Knechtges TE, Kehoe ME, et al. Comparison of adrenal vein sampling and computed tomography in the differentiation of primary aldosteronism. J Clin Endocrinol Metab. 2001;86(3):1066-71.
26. Sarlon-Bartoli G, Michel N, Taieb D, Mancini J, Gonthier C, Silhol F, et al. Adrenal venous sampling is crucial before an adrenalectomy whatever the adrenal-nodule size on computed tomography. J Hypertens. 2011;29(6):1196-202.
27. Hu J, Xu T, Shen H, Song Y, Yang J, Zhang A, et al. Accuracy of Gallium-68 Pentixafor Positron Emission Tomography–Computed Tomography for Subtyping Diagnosis of Primary Aldosteronism. JAMA Network Open. 2023;6(2):e2255609-e.
28. Lau JH, Sze WC, Reznek RH, Matson M, Sahdev A, Carpenter R, et al. A prospective evaluation of postural stimulation testing, computed tomography and adrenal vein sampling in the differential diagnosis of primary aldosteronism. Clin Endocrinol (Oxf). 2012;76(2):182-8.
29. Raman SP, Lessne M, Kawamoto S, Chen Y, Salvatori R, Prescott JD, et al. Diagnostic performance of multidetector computed tomography in distinguishing unilateral from bilateral abnormalities in primary hyperaldosteronism: comparison of multidetector computed tomography with adrenal vein sampling. J Comput Assist Tomogr. 2015;39(3):414-8.
30. Yen RF, Wu VC, Liu KL, Cheng MF, Wu YW, Chueh SC, et al. 131I-6beta-iodomethyl-19-norcholesterol SPECT/CT for primary aldosteronism patients with inconclusive adrenal venous sampling and CT results. J Nucl Med. 2009;50(10):1631-7.
31. Kim BC, Yoon HK, Park KJ, Kim GH, Pak SJ, Kwon D, et al. Diagnostic consistency between computed tomography and adrenal vein sampling of primary aldosteronism: leading to successful curative outcome after adrenalectomy; a retrospective study. Int J Surg. 2024;110(2):839-46.
32. Gkaniatsa E, Sakinis A, Palmér M, Muth A, Trimpou P, Ragnarsson O. Adrenal venous sampling in young patients with primary aldosteronism. Extravagance or irreplaceable? J Clin Endocrinol Metab. 2021;106(5):e2087-e95.
33. Sohaib SA, Peppercorn PD, Allan C, Monson JP, Grossman AB, Besser GM, et al. Primary hyperaldosteronism (Conn syndrome): MR imaging findings. Radiology. 2000;214(2):527-31.
34. Li S, Ren Y, Zhu Y, Sun H, Ma L, Tian H, et al. The potential clinical application of a lower bilateral adrenal limb width ratio (L/Rw) in patients with bilateral primary hyperaldosteronism. Endocr Pract. 2019;25(8):830-5.
35. Zhang W, Wang J, Shao M, Zhao Y, Ji H, Guo F, et al. The performance of left/right adrenal volume ratio and volume difference in predicting unilateral primary aldosteronism. Journal of Endocrinological Investigation. 2023;46(4):687-98.
36. Burton TJ, Mackenzie IS, Balan K, Koo B, Bird N, Soloviev DV, et al. Evaluation of the sensitivity and specificity of (11)C-metomidate positron emission tomography (PET)-CT

for lateralizing aldosterone secretion by Conn's adenomas. J Clin Endocrinol Metab. 2012;97(1):100-9.
37. Puar TH, Khoo CM, Tan CJ, Tong AKT, Tan MCS, Teo AE, et al. 11C-Metomidate PET-CT versus adrenal vein sampling to subtype primary aldosteronism: a prospective clinical trial. J Hypertens. 2022;40(6):1179-88.
38. Wu X, Senanayake R, Goodchild E, Bashari WA, Salsbury J, Cabrera CP, et al. [11C]metomidate PET-CT versus adrenal vein sampling for diagnosing surgically curable primary aldosteronism: a prospective, within-patient trial. Nature Medicine. 2023;29(1):190-202.
39. Soinio M, Luukkonen AK, Seppänen M, Kemppainen J, Seppänen J, Pienimäki JP, et al. Functional imaging with 11C-metomidate PET for subtype diagnosis in primary aldosteronism. Eur J Endocrinol. 2020;183(6):539-50.
40. Ding J, Zhang Y, Wen J, Zhang H, Wang H, Luo Y, et al. Imaging CXCR4 expression in patients with suspected primary hyperaldosteronism. Eur J Nucl Med Mol Imaging. 2020;47(11):2656-65.
41. Yin X, Ai K, Luo J, Liu W, Ma X, Zhou L, et al. A comparison of the performance of (68)Ga-Pentixafor PET/CT versus adrenal vein sampling for subtype diagnosis in primary aldosteronism. Front Endocrinol (Lausanne). 2024;15:1291775.
42. Gross MD, Shapiro B, Freitas JE. Limited significance of asymmetric adrenal visualization on dexamethasone-suppression scintigraphy. J Nucl Med. 1985;26(1):43-8.
43. Saiga A, Yokota H, Nagano H, Sawada K, Kubota Y, Wada T, et al. 131I-6β-iodomethyl-19-norcholesterol adrenal scintigraphy as an alternative to adrenal venous sampling in differentiating aldosterone-producing adenoma from bilateral idiopathic hyperaldosteronism. Nucl Med Commun. 2020;41(12):1226-33.
